# A novel transcriptional cascade is involved in Fzr-mediated endoreplication

**DOI:** 10.1093/nar/gkaa158

**Published:** 2020-03-17

**Authors:** Wenliang Qian, Zheng Li, Wei Song, Tujing Zhao, Weina Wang, Jian Peng, Ling Wei, Qingyou Xia, Daojun Cheng

**Affiliations:** 1 State Key Laboratory of Silkworm Genome Biology, Biological Science Research Center, Southwest University, Chongqing 400715, China; 2 Chongqing Key Laboratory of Sericultural Science, Chongqing engineering and technology research center for novel silk materials, Southwest University, Chongqing 400715, China; 3 Medical Research Institute, Wuhan University, Wuhan 430071, China; 4 Department of Genetics, Harvard Medical School, Boston, MA 02115, USA; 5 School of Life Science, Southwest University, Chongqing 400715, China

## Abstract

Endoreplication, known as endocycle, is a variant of the cell cycle that differs from mitosis and occurs in specific tissues of different organisms. Endoreplicating cells generally undergo multiple rounds of genome replication without chromosome segregation. Previous studies demonstrated that *Drosophila* fizzy-related protein (Fzr) and its mammalian homolog Cdh1 function as key regulators of endoreplication entrance by activating the anaphase-promoting complex/cyclosome to initiate the ubiquitination and subsequent degradation of cell cycle factors such as Cyclin B (CycB). However, the molecular mechanism underlying Fzr-mediated endoreplication is not completely understood. In this study, we demonstrated that the transcription factor Myc acts downstream of Fzr during endoreplication in *Drosophila* salivary gland. Mechanistically, Fzr interacts with chromatin-associated histone H2B to enhance H2B ubiquitination in the *Myc* promoter and promotes *Myc* transcription. In addition to negatively regulating *CycB* transcription, the Fzr-ubiquitinated H2B (H2Bub)-Myc signaling cascade also positively regulates the transcription of the *MCM6* gene that is involved in DNA replication by directly binding to specific motifs within their promoters. We further found that the Fzr-H2Bub-Myc signaling cascade regulating endoreplication progression is conserved between insects and mammalian cells. Altogether, our work uncovers a novel transcriptional cascade that is involved in Fzr-mediated endoreplication.

## INTRODUCTION

Endoreplication, also called endocycle, is a unique variant of the cell cycle in which cells undergo multiple rounds of genome replication and lacks mitosis and chromosome segregation, resulting in polyploidy (1–3). Endoreplication frequently occurs in specific tissues or cells of animals and plants, such as *Drosophila* salivary gland and follicular epithelium, silkworm silk gland, and mammalian trophoblast giant cell. Salivary gland and silk gland are developmentally formed by the cell division cycle called mitosis at the late stage of embryogenesis and grow into large organs via approximately 10 and 17–19 rounds of endocycles, respectively, in each cell during the larval period (4,5). The DNA content subsequently increases to 1350 copies (C) in a single cell of the salivary gland and is increased by ∼400 000 times in the silk gland (4–6). Mammalian trophoblast giant cells endoreplicate and produce polytene chromosomes with DNA contents of up to 512 C (7). Generally, endoreplication is essential for tissue-specific functions or adaptation to environmental stress (3).

The progression of endoreplication involves three key events: the mitotic-to-endocycle transition, oscillation of DNA re-replication, and progression of DNA replication (2). Increasing evidence has demonstrated that fizzy-related protein (Fzr; also known as Cdh1 in mammals), which contains seven WD40 domains, is essential for the mitotic-to-endocycle transition and the oscillation of DNA re-replication in both *Drosophila* and mammals (2,3,8–10). In *Drosophila*, *Fzr* transcription is upregulated in epidermal cells after mitosis 16 during embryogenesis when cells exit from the mitotic cycle, accompanied by a significant elimination of mitotic cyclins (8,11,12). Disruption of *Fzr* expression results in a failure of the mitotic-to-endocycle transition in both the larval salivary gland and follicle cells of the adult ovary (8,9,13).

Fzr has been previously shown to activate the anaphase-promoting complex/cyclosome (APC/C), which has E3 ubiquitin ligase activity; APC/C activation in turn promotes ubiquitin-mediated cyclic degradation of cell cycle factors, including Cyclin B (CycB) at telophase and G1 phase in mitotic cells as well as Geminin during G1-like phase in endoreplicating cells (2,3,14–16). Data from endocycling *Drosophila* salivary gland indicates that the oscillation of APC/C^Fzr^ activity is regulated by periodic Cyclin E/Cyclin-dependent kinase 2 (CycE/Cdk2) activity and is essential for DNA re-replication in endocycle progression (2,3). Low CycE/Cdk2 activity during early G1-like phase activates APC/C^Fzr^, and subsequent Geminin degradation triggers the assembly of prereplication complexes (preRCs) at DNA replication origins, whereas high CycE/Cdk2 activity during late G1-like phase inactivates APC/C^Fzr^ to initiate S phase entry and DNA re-replication in the presence of Geminin (3,17–20). Importantly, CycB is required for mitosis entry in mitotic cells (21–23). Previous studies have reported that not only CycB protein is absent in endoreplicating cells of both *Drosophila* salivary gland and silkworm silk gland (8,24–26), but also *CycB* mRNA cannot be detected in endoreplicating cells (26,27), indicating that transcriptional repression of the *CycB* gene is likely essential for endoreplication. Given that Fzr functions as a key regulator of mitotic-to-endocycle transition and endoreplication control, we therefore hypothesized that the loss of CycB in endoreplicating cells may be due to potential transcriptional repression by the Fzr-mediated pathway.

DNA replication is a common process in both endocycling and mitotic cells, and its control is conserved. Generally, DNA replication is initiated at origins of replication by binding with the origin recognition complex (ORC) along the chromosomes, and subsequently, several key factors are recruited before S phase to form preRCs, which permit the initiation of DNA replication (3,28,29). Notably, mini-chromosome maintenance proteins 2–7 (MCM2–7) are identified as preRC components and interact with each other to form a stable heterohexamer, which functions as a DNA helicase to melt the DNA double helix (30–32). Upregulating the expression of *MCM4* and *MCM7* promotes DNA replication and is essential for polyploidy in endocycling cells, while knockdown of these *MCM* genes blocks DNA replication and causes reduced ploidy (33). DNA replication depends on CycE/Cdk2 activity, and an inactive kinase complex is required for preRC assembly (20,34). Given the regulatory roles of MCMs and Fzr in preRC assembly during DNA synthesis, we sought to determine whether Fzr is also involved in modulating MCM functions.

In this study, we identified a novel transcriptional cascade involved in Fzr-mediated regulation of endoreplication in *Drosophila* salivary gland. Salivary gland-specific knockdown of *Fzr* expression blocked DNA replication during endoreplication and resulted in both the appearance of *CycB* mRNA expression and the absence of *MCM6* mRNA expression. Mechanistically, we showed that Fzr interacts with chromatin-associated histone 2B (H2B) and enhances the level of ubiquitinated H2B (H2Bub) in the promoter region of the *Myc* gene to promote its transcription. Myc in turn negatively and positively regulates the transcription of *CycB* and *MCM6*, respectively, by directly binding to their promoters. Finally, we confirmed that the Fzr-H2Bub-Myc signaling axis is conserved in insects and mammalian cells. Altogether, our findings provide novel insights into the mechanism underlying Fzr regulation of endoreplication progression.

## MATERIALS AND METHODS

### *Drosophila* stocks

All *Drosophila* stocks were reared at 25°C under standard feeding conditions, and the living environment was maintained at 65% humidity with a cycle of 12-h light:12-h dark, as previously described (35). The following RNAi lines were obtained from the Vienna *Drosophila* Resources Center (VDRC): *UAS*-*Fzr* RNAi (#25550) (36), *UAS*-*Myc* RNAi (#2947) (37) and the VDRC control (#60000). The stocks from TsingHua Fly Center include: *UAS*-*Fzr* RNAi (#TH201500745.S), *UAS*-*Myc* RNAi (#THU5827) (37), *UAS*-*Apc1* RNAi (#TH201500835.S), *UAS*-*Apc2* RNAi (#THU3480), *UAS*-*Apc3* RNAi (#THU3449), *UAS*-*Apc6* RNAi (#TH201500101.S), *UAS*-*Apc10* RNAi (#THU3532) and the related control (#TB00072) (38). *Nubbin*-Gal4 (#42699), *UAS-Myc* (#9674) (39) and *UAS*-*MCM6* RNAi (#41842) were obtained from the Bloomington *Drosophila* Stock Center (BDSC). The *UAS*-*Fzr* (#F000893) line was obtained from FlyORF (40). The *UAS*-*CycB* RNAi (#3510R-1) line was obtained from Fly Stocks of National Institute of Genetics (NIG-FLY) (41). *Sg*-Gal4 that is used to specifically drive gene expression in *Drosophila* salivary glands (42), *hsFlp*; *act*>*CD2*>*Gal4*, *UAS-nlsGFP*/*Cyo* (43), and w^1118^ as wild type were generously gifted from Prof. Norbert Perrimon. The RNAi of the *MCM6* gene was conducted at 29°C for enhancing knockdown efficiency and other genetic manipulation experiments were conducted at 25°C. For developmental timing in *Drosophila*, six to eight fertilized female flies were allowed to lay eggs at 25°C for 6 h in a vial containing standard food and were then moved. These eggs of the control generally develop to pupariation at ∼120 h after egg laying (AEL).

### RNA extraction and quantitative real-time RT-PCR (RT-qPCR)

Total RNA samples were prepared from *Drosophila* salivary gland or cultured *Drosophila* S2 cells using Trizol reagent (Invitrogen). According to the manufacturer's protocol of the M-MLV Reverse Transcriptase Kit (Promega), 2 μg of total RNA was used for cDNA synthesis. RT-qPCR was performed with a SYBR Premix ExTaq Kit (Takara) and a qTower 2.2 Real-time PCR Detection System (Jena). The α-tubulin at 84B gene (*α-tub84B*; NM_057424) was used as the internal control. All experiments were independently performed with three biological replicates, and the relative mRNA expression levels were calculated using the 2^−ΔΔCT^ method. All primers used for RT-qPCR are listed in Supplementary Table S1.

### RNA sequencing (RNA-seq) and data analysis

The open reading frame (ORF) sequence of the *Drosophila Fzr* gene was subcloned into pMT-V5-HisA vector for gene overexpression in *Drosophila* S2 cells. At 48 h after vector transfection into S2 cells following an induction of 500 μM CuSO_4_, total RNA samples were separately prepared from *Fzr*-overexpressed S2 cells and the control. Three biological replicates were performed. Each of total RNA samples was then sequenced on a HiSeq 2500 platform (Novogene). All raw sequence reads were mapped to the *Drosophila* genome assembly BDGP6 by using the Hisat2 software. Gene expression levels were evaluated by using the FPKM (fragments per kilobase of transcript sequence per millions base pairs sequenced) values. Differential expression analysis was performed by using the DESeq2 package (44). The resulting *P*-values were adjusted by using the Benjamini and Hochberg's approach for controlling the false discovery rate. Differentially expressed genes were determined with the threshold criterion of an adjusted *P* value of <0.05. All raw data in this study have been uploaded to the Sequence Read Archive of the National Center for Biotechnology Information (NCBI) database (accession number: PRJNA509304).

### DNA quantification

The C-value of the genome in *Drosophila* salivary gland cells was quantified based on DAPI fluorescence intensity (20). In summary, the salivary glands used in different experiments were dissected at the indicated time and stained with DAPI (1:1000; Thermo Fisher Scientific). The salivary glands of the wild-type strain were used as the internal control. Z-stack images of fluorescence signals for each sample were acquired at 60× with confocal microscope (Olympus Fv1000) under the same laser intensity. The integrated DAPI fluorescence intensity was subtracted for C-value measurement. The C-value of the control salivary gland cell is set as 1350 C (4). For DNA content quantification, 50 salivary glands from wandering *Drosophila* larvae were dissected for genome DNA subtraction. The DNA content was quantified spectrophotometrically using an Agilent 2100 Bioanalyzer System (Agilent).

### EdU and BrdU staining

EdU staining was performed by using a commercial Cell Light EdU Apollo 567 *in vitro* Kit (Ribobio) according to the previously described procedure (35). For EdU labeling, the salivary glands dissected from *Drosophila* larvae at 96 h AEL were cultured with 100 μg/ml EdU *in vitro* for 2 h at room temperature. The cultured samples were fixed with PBS containing 4% paraformaldehyde for 30 min and then incubated with Apollo dye for 30 min. Subsequently, the tissues were stained with DAPI and mounted in Vectashield buffer. For BrdU staining, the salivary glands were cultured with 100 μg/ml BrdU for 2 h at room temperature. Following fixation, the samples were treated with 2 N HCl for 30 min. The tissues were subsequently incubated with mouse anti-BrdU antibody (1:50; Roche) and with anti-mouse-IgG-Cy3 (1:1000; Roche) as the secondary antibody for the detection of incorporated BrdU. Fluorescence signals were captured by confocal microscopy (Zeiss LSM 880 and Olympus Fv1000).

### Immunostaining

Immunostaining assay was performed in the salivary glands and cultured cells as previously described (35,45). Briefly, fixed cells or tissues were washed three times with PBST buffer (1× PBS including 0.3% Triton-X 100) and then stained at 4°C overnight with primary antibodies at the following dilutions: goat anti-*Drosophila* Fzr (1:50; Santa Cruz), rabbit anti-*Drosophila* Myc (1:50; Santa Cruz), goat anti-human Fzr (1:50; Santa Cruz), mouse anti-human Myc (1:50; Santa Cruz), mouse anti-CycB (1:200; DSHB), rabbit anti-MCM6 (1:200; Zoonbio Biotechnology) and mouse anti-Myc tag (1:200; Sigma). After washing three times with PBST buffer, the samples were incubated with the corresponding Alexa Fluor-conjugated secondary antibodies (Life Technologies). DAPI (1:1000; Thermo Fisher Scientific) was used for nuclear labeling, while Alexa Fluor-conjugated phalloidin (1:1000; Life Technologies) was used for Actin staining. Finally, after washing with PBS three times, the cells or tissues were mounted in Vectashield mounting buffer and the fluorescence signals were captured by confocal microscope (Zeiss LSM 880 and Olympus Fv1000).

### Fluorescence *in situ* hybridization

Digoxygenin (DIG)-labeled *DmCycB* probes were synthesized for fluorescence *in situ* hybridization. According to a previously described procedure (35), the isolated salivary glands were fixed with 4% paraformaldehyde and then permeabilized in PBST buffer for 30 min. Following the prehybridization process in hybridization buffer (5× SSC and 50% deionized formamide), samples were incubated with DIG-labeled probes for 12 h at 56°C. After a series of washes to significantly decrease background, the salivary glands were incubated with rabbit anti-DIG antibody (1:1000; Invitrogen) for 2 h and then with an anti-rabbit Alexa Fluor 594-conjugated antibody (1:1000; Life Technologies). DAPI (1:1000; Thermo Fisher Scientific) was used for nuclear labeling. The signals were captured by confocal microscope (Olympus Fv1000). The primers used to prepare the DIG probes are listed in Supplementary Table S1.

### Western blotting

Total proteins were isolated from *Drosophila* tissues and the cultured cells with different treatments, and the protein concentration in the lysates was quantified using Bio-Rad protein assay reagent. Equal amounts of total protein were subjected to western blotting. The antibodies and dilutions used in the study were as follows: goat anti-*Drosophila* Fzr (1:1000; Santa Cruz), rabbit anti-*Drosophila* Myc (1:1000; Santa Cruz), goat anti-human Fzr (1:1000; Santa Cruz), mouse anti-human Myc (1:1000; Santa Cruz), mouse anti-*Drosophila* CycB (1:5000; DSHB), rabbit anti-human CycB (1:5000; Cell Signaling), mouse anti-H2B (1:10 000; Beyotime), rabbit anti-H2Bub (1:20 000; Cell Signaling), mouse anti-V5 (1:5000; Abcam), rabbit anti-Flag (1:5000; Sigma), mouse anti-Myc tag (1:5000; Sigma), rabbit anti-MCM6 (1:5000; Zoonbio Biotechnology), and mouse anti-tubulin (1:10 000; Beyotime). The following secondary antibodies were used, including HRP-conjugated goat anti-rabbit (1:10 000; Beyotime), goat anti-mouse (1:10 000; Beyotime), and donkey anti-goat (1:10 000; Beyotime).

### Co-immunoprecipitation (Co-IP) experiment and liquid chromatography–tandem mass spectrometry (LC–MS/MS) analysis

For Co-IP analysis with total proteins, the cells co-overexpressing *Drosophila Fzr* and *H2B* were lysed in NP-40 lysis buffer (Beyotime) containing 1 mM Protease Inhibitor Cocktail (Sigma) on ice for 10 min. After centrifugation at low temperature, the supernatants were collected. For Co-IP analysis with nucleoproteins, the nuclei of *Drosophila* S2 cells co-overexpressing *Fzr* and other molecules were extracted by NE-PER nuclear and cytoplasmic extraction kit (Thermo Scientific Pierce) and were subsequently digested by micrococcal nuclease (Cell Signaling) into short fragment or even into mononucleosome. Next, according to a previously described procure (45), the nuclear extracts were incubated with specific antibodies crosslinked with protein A/G magnetic Dynabeads (Invitrogen) containing 1 mM Protease Inhibitor Cocktail (Sigma) under gentle rotation at 4°C for 6 h. Beads were washed for three times with NP-40 lysis buffer containing 1 mM Protease Inhibitor Cocktail and then eluted with SDT buffer containing 4% (w/v) SDS and 100 mM Tris/HCl (pH 7.4) to capture target proteins. The eluted samples were detected by western blotting. In addition, to identify potential partners interacting with Fzr, human HEK293-FT cells overexpressing human *Fzr* were lysed in NP-40 lysis buffer containing 1 mM Protease Inhibitor Cocktail (Sigma) on ice, and the supernatants were collected for Co-IP with an antibody against human Fzr. The eluted samples were subjected to LC-MS/MS analysis by Shanghai Applied Protein Technology.

### Translating ribosome affinity purification (TRAP) analysis

According to the principle of the TRAP approach (46,47), the salivary glands of *Fzr* knockdown animals and the related control were isolated at 84 h AEL, 96 h AEL and 120 h AEL, respectively. A set of salivary glands was used to isolate total RNA and 1 μg RNA was reversed for input sample. Another set of salivary glands was cultured in cycloheximide (Sigma) for 2 h and then lysed in NP-40 lysis buffer containing RNase inhibitor RNasin (Promega); The ribosome–mRNA complexes were subsequently affinity purified with a Co-IP assay using specific antibody (Beyotime) against RPS20, a component of the 40S ribosomal subunit, and the mRNAs in the IP products were then isolated with MicroElute Total RNA kit (Omega) for further RT-qPCR assays. The input samples were used as the internal control. The primers are listed in Supplementary Table S1.

### GST pull-down assay

The full-length ORF of *Drosophila Fzr* and *H2B* were subcloned into the pGEX and pCold-SUMO vectors to express GST-tagged Fzr and SUMO-His-tagged H2B, respectively. Following a standard prokaryotic expression and protein purification, the purified GST-Fzr and SUMO-His-H2B were used for further GST pull-down assay. Briefly, Glutathione agarose beads coated with either GST or GST-Fzr recombinant protein were mixed with SUMO-His or SUMO-His-H2B. After an incubation under shaking for 6 h at 4°C, the beads were collected and then eluted with SDT buffer containing 4% (w/v) SDS and 100 mM Tris/HCl (pH 7.4) to capture target proteins. The products were subjected to western blot assays to evaluate the interaction between Fzr and H2B. The primers are listed in Supplementary Table S1.

### *In vivo* ubiquitination assay

To verify Fzr-mediated H2B ubiquitination, we performed *in vivo* ubiquitination experiments as previously described (48). Briefly, HA-tagged ubiquitin or HA-tagged ubiquitin-K0 (all lysines are mutated to arginines and it only mediates mono-ubiquitination), Myc-tagged H2B, and Flag-tagged Fzr overexpression plasmids were co-transfected into *Drosophila* S2 cells. At 48 h after transfection, the cells were treated with proteasome inhibitor MG132 (50 μM; Selleck) for 6 h and the nuclear extracts were harvested by NE-PER nuclear and cytoplasmic extraction kit (Thermo Scientific Pierce) according to the instruction. Following Co-IP with the indicated antibodies, the products were subjected to western blot assays to detect the H2B ubiquitination patterns.

### Chromatin immunoprecipitation (ChIP)

Chromatin immunoprecipitation experiment following quantitative PCR (ChIP-qPCR) or basic PCR (ChIP-PCR) assay was performed to evaluate the level of H2Bub in the *Drosophila Myc* promoter and the direct binding of Myc within the promoters of either *CycB* or *MCM6*. According to the manufacturer's instructions for the EZ-ChIP Immunoprecipitation Kit (Millipore) and a previous study (45), the cells overexpressing *Drosophila Fzr* or *Myc* were fixed with 37% formaldehyde to crosslink chromatin and the binding proteins, and then quenched in 125 mM glycine. After washing for two times, the cells were lysed in NP-40 lysis buffer (Beyotime) containing 1 mM Protease Inhibitor Cocktail (Sigma) on ice for 10 min. Subsequently, the samples were sonicated to shear the DNA into fragments of 200–1000 bp in length. The chromatin-protein complexes were separately immunoprecipitated with specific antibodies. The purified DNA fragments enriched in the eluted immunoprecipitants were evaluated by quantitative PCR or basic PCR reaction with primer pairs covering potential promoter regions of the selected gene. The primers used for ChIP-qPCR or ChIP-PCR are listed in Supplementary Table S1.

### Dual luciferase reporter assay

By using the MatInspector program (http://www.genomatix.de/), we predicted the E-box sequences for Myc binding within potential promoter regions and coding sequence regions of the *CycB* and *MCM6* genes. According to our prediction and *Drosophila* Myc ChIP-seq data in previous report (49), we carried out a dual luciferase reporter assay to further determine Myc regulation on the transcriptions of *CycB* and *MCM6*. The ORF sequence of the *Myc* gene was subcloned into the pMT-V5-HisA vector for gene overexpression, while truncated fragments of the promoters with or without E-box sequences were subcloned into the pGL3-Basic vector to drive the expression of the firefly luciferase gene. These two types of plasmids were co-transfected into *Drosophila* S2 cells. At 48 h after transient transfection following an induction of 500 μM CuSO_4_, the cells were collected, and a dual luciferase assay was performed as described previously (45). The RL-TK vector containing the Renilla luciferase gene was used as the internal control. The primers used in the dual luciferase assay are listed in Supplementary Table S1.

### Electrophoretic mobility shift assay (EMSA)

According to the predicted E-box for Myc binding within the promoters of the *CycB* and *MCM6* genes, DNA probes targeting the predicted E-box motifs were labeled with biotin at the 5′ end. Following the instructions of the EMSA/Gel-Shift Kit (Beyotime), 10 μL of binding reaction systems containing 100 nM probes and different amounts of extracted nucleoproteins from *Myc*-overexpressing *Drosophila* S2 cells (1, 3, 5 and 7 μg) were prepared. For the competition assay, 7 μg of extracted nucleoproteins were first incubated with a 1-fold, 5-fold, 25-fold and 50-fold molar excess of the unlabeled probes before the biotin-labeled probes were added. For the antibody-dependent EMSA, 7 μg of extracted nucleoproteins were first incubated with 2 μg of a specific anti-Myc antibody. All reaction systems were electrophoresed on 5% (w/v) polyacrylamide gels in TBE buffer (45 mM Tris borate and 1 mM EDTA, pH 8.3). The primers used for EMSA are listed in Supplementary Table S1.

### Statistical analysis

Data are presented as the mean ± SE of three independent biological replicates. Statistical significance (*P*-value) was analyzed by an unpaired, two-tailed Student's t-test. Statistical significance is denoted as follows: *P < 0.05, **P < 0.01 and ***P < 0.001.

## RESULTS

### Changes of *Fzr* expression disrupt endoreplication progression in *Drosophila* salivary gland and alter *CycB* transcription

**Figure 1.**
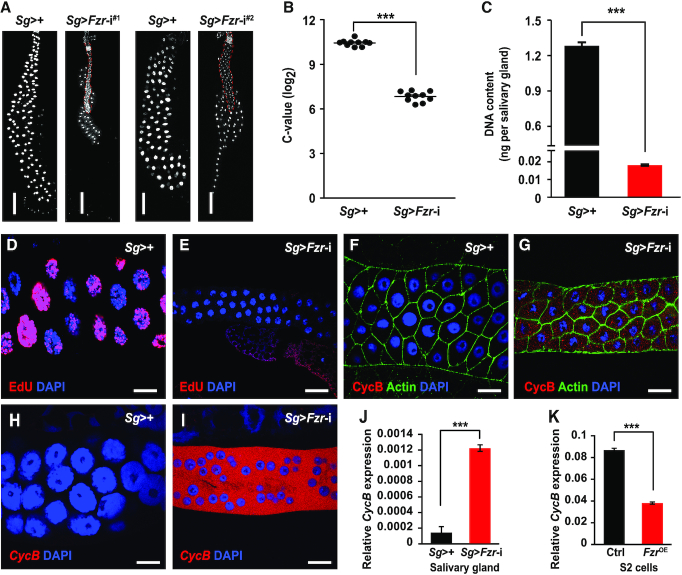
Specific knockdown of *Fzr* expression in *Drosophila* salivary gland blocks endoreplication and results in the initiation of *CycB* transcription. (**A**) RNAi-mediated *Fzr* knockdown driven by salivary gland-specific *Sg*-Gal4 resulted in a significant reduction in the gland size. At 120 h AEL, salivary glands from control and *Fzr* knockdown *Drosophila* were dissected and stained with DAPI. Two *Fzr* RNAi lines targeting different sequences were used in this experiment. *Fzr*-i^#1^: *UAS-Fzr* RNAi line from VDRC (#V25550); *Fzr*-i^#2^: *UAS-Fzr* RNAi line from TsingHua Fly Center (#TH2015000745.S). Scale bar, 180 μm. (**B**) C-value was quantified by DAPI fluorescence. The salivary glands from wandering *Drosophila* larvae at 120 h AEL were fixed and stained with DAPI. The integrated DAPI intensity was used to measure the DNA content. (**C**) Quantification of the DNA content in the salivary glands of *Drosophila* larvae at 120 h AEL. DNA was extracted from the indicated salivary gland cells and quantified via absorbance analysis. (**D**, **E**) EdU staining of DNA replication. In the salivary glands of larvae at 96 h AEL, the nuclei of most endocycling cells in the salivary glands as control could be strongly stained with EdU, indicating that DNA synthesis is ongoing. However, no replication signals were detected in salivary gland cells with *Fzr* knockdown. Scale bar, 30 μm. (**F–J**) *Fzr* knockdown in the salivary glands causes an accumulation of the CycB protein (F, G) and an appearance of the *CycB* mRNA (H–J) at 96 h AEL. The mRNA level was measured by fluorescence *in situ* hybridization (H, I) and RT-qPCR(J). Scale bar, 30 μm. **(K)***Fzr* overexpression in *Drosophila* S2 cells significantly downregulated the transcription of the *CycB* gene. Data are presented as mean ± SE (error bars). For the significance test: ***P < 0.001 versus control. OE, overexpression. AEL, after egg laying.

To better understand the regulatory mechanism underlying Fzr regulation of endoreplication in *Drosophila* salivary gland, we first performed RNAi-mediated knockdown of the *Fzr* gene in the salivary gland by using the *Sg*-Gal4 driver, which is specifically active in the salivary gland (42). Salivary gland-specific knockdown of the *Fzr* gene mediated by two RNAi lines targeting distinct sequences of the *Fzr* gene significantly reduced the gland size (Figure 1A and Supplementary Figure S1A) and DNA content (Figure 1B-C and Supplementary Figure S1B-C). Conversely, *Fzr* overexpression driven by *Nubbin-*Gal4 in wing disc cells with mitotic cycle resulted in an increase in both the nuclear size and DNA content as well as the formation of abnormally widened posterior compartments (Supplementary Figure S1D-E). Similarly, *Fzr* overexpression in *Drosophila* S2 cells enlarged the size of both the nuclei and the cells (Supplementary Figure S1F). Moreover, ongoing DNA synthesis was detected in the salivary gland by using EdU or BrdU staining (Figure 1D and Supplementary Figure S1G), but *Fzr* knockdown (based on the *Fzr-*i^#1^ line, hereafter the same) abrogated DNA synthesis (Figure 1E and Supplementary Figure S1G). Taken together, conditional loss- and gain-of-function analyses further indicate that Fzr is essential for the mitotic-to-endocycle transition and DNA replication in salivary gland cells.

The cyclin protein CycB is essential for M phase entry during mitotic cell cycles, and its ubiquitin-dependent degradation driven by Fzr-activated APC/C is necessary for the G1/S transition (21–23). The CycB protein is absent in endoreplicating *Drosophila* salivary glands and silkworm silk glands (8,26), indicating that the absence of CycB is likely required for endocycle progression. To uncover the mechanism underlying the absence of CycB in the *Drosophila* salivary glands, we investigated the effects of *Fzr* expression changes on *CycB* expression at the protein and mRNA levels. Strikingly, the CycB protein could not be detected in the salivary glands as control (Figure 1F), but salivary gland-specific *Fzr* knockdown caused an accumulation of the CycB protein (Figure 1G). Similarly, no *CycB* transcript was detected in the salivary glands as control (Figure 1H), whereas *Fzr* knockdown in the salivary glands obviously elevated *CycB* transcription (Figure 1I and J). Moreover, *Fzr* overexpression in *Drosophila* S2 cells inhibited *CycB* mRNA transcription and protein expression (Figure 1K and Supplementary Figure S1H). These data indicate that Fzr is likely involved in inhibiting *CycB* expression in endoreplicating salivary glands at the transcriptional level.

Given that Fzr-mediated signaling can degrade CycB (24,25), we further determined whether or not the increased CycB proteins following *Fzr* knockdown in the salivary glands were driven by the increased *CycB* mRNA. First, TRAP analysis following RT-qPCR analysis showed that the increased *CycB* mRNA was highly translated at 84 h AEL in the salivary glands with *Fzr* knockdown and exhibited a moderate translation at 96 h AEL and 120 h AEL (Supplementary Figure S2A). In addition, western blotting revealed that the CycB proteins were also accumulated from 84 h AEL to 96 h AEL, and maintained a relative constant level at 120 h AEL (Supplementary Figure S2B). Furthermore, we observed that the accumulation of both the *CycB* mRNA and the CycB protein in the salivary glands with *Fzr* knockdown was abrogated by *CycB* silencing (Supplementary Figure S2C and D–F). Taken together, these data indicate that the accumulation of the *CycB* mRNA in the salivary glands following *Fzr* knockdown is a main driving force behind the increased CycB proteins.

### Myc functions as a downstream effector of Fzr signaling in endoreplication progression and inhibits *CycB* transcription

**Figure 2.**
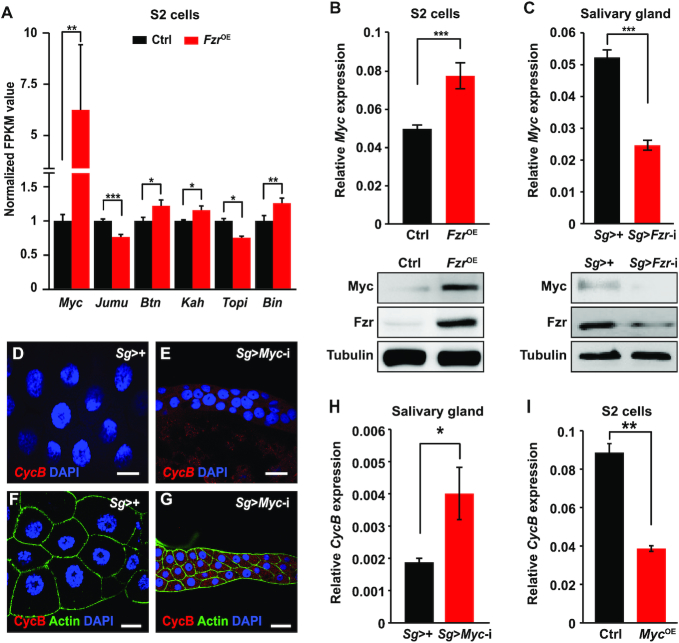
*Fzr* expression changes affect the transcription of transcription factor gene *Myc* and Myc is required for inhibition of *CycB* transcription in *Drosophila* salivary gland. (**A**) Transcriptome analysis of expression changes of several transcription factor genes following *Fzr* overexpression in *Drosophila* S2 cells. Normalized FPKM value was used for measuring relative expression level. *Myc* was upregulated following *Fzr* overexpression. Data are presented as mean + SE (error bars). For the significance test: *P < 0.05, **P < 0.01, ***P < 0.001 versus control. (**B**) *Fzr* overexpression in S2 cells promoted mRNA transcription and protein expression of the *Myc* gene. (**C**) *Fzr* knockdown in the salivary glands reduced mRNA transcription and protein expression of the *Myc* gene at 96 h AEL. (**D–H**) *Myc* knockdown in the salivary glands causes an accumulation of the *CycB* mRNA (D, E and H) and the CycB protein (F, G) at 96 h AEL. (**I**) *Myc* overexpression in *Drosophila* S2 cells significantly downregulated the transcription of the *CycB* gene. Data are presented as mean ± SE (error bars). For the significance test: *P < 0.05, **P < 0.01, ***P < 0.001 versus control. OE, overexpression. AEL, after egg laying. Scale bar, 30 μm.

Fzr contains seven WD40 domains that mediate protein-protein interactions and is excluded from the family of transcription factors (22,24,25). To identify transcription factors that are involved in regulating the transcription of the *CycB* gene as downstream effector of Fzr signaling, we conducted RNA-seq-based transcriptome analysis of *Drosophila* S2 cells with *Fzr* overexpression. The results revealed that compared to the control, a total of 38 and 39 genes were upregulated and downregulated in *Fzr*-overexpressing S2 cells, respectively (Supplementary Figure S3A and Supplementary Table S2), and the transcription factor gene *Myc* was highly upregulated following *Fzr* overexpression (Figure 2A). Further RT-qPCR and western blotting confirmed that *Fzr* overexpression enhanced *Myc* expression in either *Drosophila* S2 cells or wing disc (Figure 2B and Supplementary Figure S3B). In contrast, *Fzr* knockdown in the salivary glands reduced *Myc* expression (Figure 2C and Supplementary Figure S3C–D′).

We further performed salivary gland-specific loss- and gain-of-function analyses of *Drosophila Myc* and found that Myc phenocopied the effects of Fzr on endoreplication. First, *Myc* knockdown in the salivary glands dramatically reduced gland size, the size of the cell and nucleus, and DNA content (Supplementary Figure S4A–E). EdU or BrdU staining revealed no DNA replication following either *Myc* knockdown (Supplementary Figure S4F and G) or clonal *Myc* silencing (Supplementary Figure S4H and I). Second, we carried out salivary gland-specific *Myc* overexpression and observed that at 120 h AEL (the beginning of the prepupal stage), DNA replication was terminated in salivary gland cells as control and these cells could not be stained with EdU, whereas salivary gland cells with *Myc* overexpression could still be labeled with EdU (Supplementary Figure S4J–M), confirming that *Myc* overexpression enhances DNA replication. Third, similar to the effects of Fzr on *CycB* expression, *Myc* knockdown in the salivary glands also induced the accumulation of both *CycB* mRNA and CycB protein (Figure 2D–H). However, *Myc* overexpression in *Drosophila* S2 cells inhibited the transcription and protein expression of *CycB* (Figure 2I and Supplementary Figure S4N).

**Figure 3.**
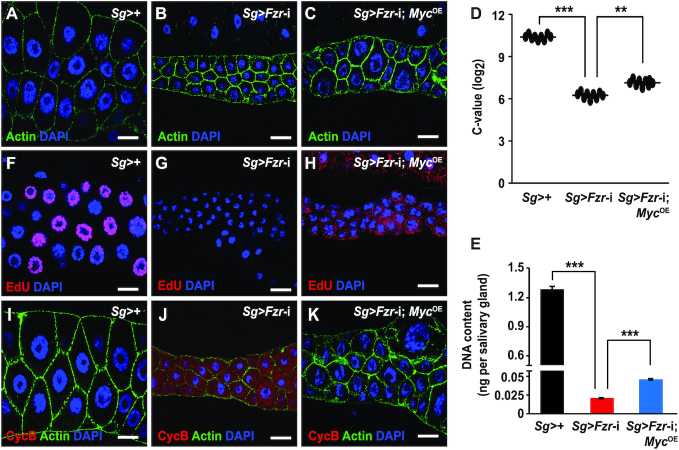
Myc functions as a downstream effector of Fzr modulation during endoreplication in *Drosophila* salivary gland. (**A–H**) Epistasis analysis revealed that *Myc* overexpression in the salivary glands moderately rescued the effects of *Fzr* knockdown on gland size (A–C), C-value (D), and DNA content (E) at 120 h AEL as well as DNA replication (F–H) at 96 h AEL. (**I–K**) *Fzr* knockdown-induced CycB expression in the salivary glands was abrogated by salivary gland-specific *Myc* overexpression at 96 h AEL. Data are presented as mean ± SE (error bars). For the significance test: **P < 0.01, ***P < 0.001 versus control. OE, overexpression. AEL, after egg laying. Scale bar, 30 μm.

We next evaluated the epistatic relationship between Fzr and Myc in the regulation of endoreplication in the salivary glands. Our results showed that the reduced nuclear size caused by salivary gland-specific *Fzr* knockdown was partially rescued by *Myc* overexpression in salivary gland cells (Figure 3A–C). In addition, compared to *Fzr* knockdown alone in salivary gland cells, *Myc* overexpression in salivary gland cells with *Fzr* knockdown caused a two-fold increase in DNA content (Figure 3D and E); reinitiation of DNA replication, as evidenced by EdU staining (Figure 3F–H); and abrogation of CycB protein accumulation (Figure 3I–K). These data, combined with our finding about the positive regulation of Fzr on *Myc* transcription, indicate that Myc functions as a downstream effector of Fzr. Taken together, it is noteworthy that *Myc* overexpression only partially restores the *Fzr* knockdown-induced changes in the nuclear and cell size as well as the DNA content in endoreplicating salivary gland cells (Figure 3A–E), suggesting that key transcriptional regulators downstream of Fzr signaling may be more than Myc in the control of salivary gland endoreplication.

### Fzr mediates histone H2B ubiquitination within the promoter of the *Myc* gene

**Figure 4.**
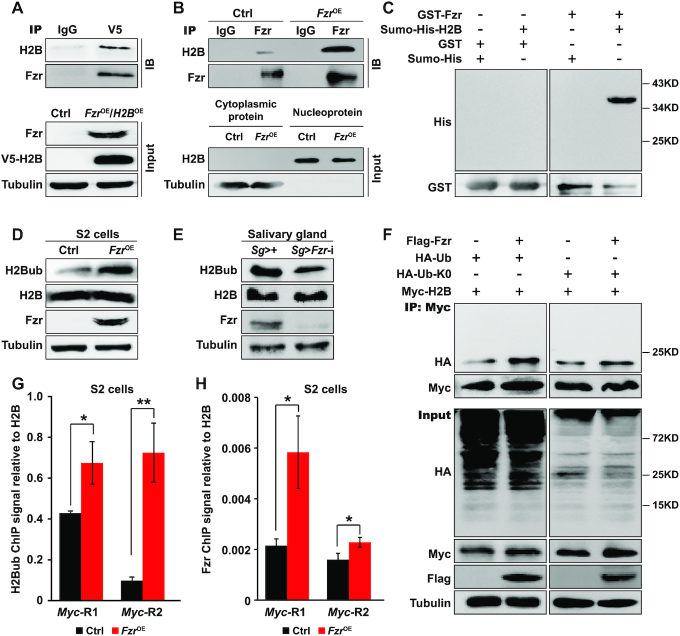
Fzr enhances H2B ubiquitination within the promoter of the *Myc* gene. (**A**) Co-IP analysis revealed that Fzr interacts with H2B. (**B**) Following nucleus isolation, micrococcal nuclease digestion, and nucleoprotein extraction, Co-IP experiments with nucleoproteins were performed and then confirmed the interaction between Fzr and chromatin-associated H2B. **(C)** GST-pull down assay showed a direct interaction between Fzr and H2B. (**D, E**) Fzr promotes H2B ubiquitination. The level of ubiquitinated H2B (H2Bub) was increased following *Fzr* overexpression in *Drosophila* S2 cells (D) and was decreased after *Fzr* knockdown in the salivary glands at 96 h AEL (E). (**F**) An *in vivo* ubiquitination assay in S2 cells confirmed that Fzr promotes H2B mono-ubiquitination. Ub-K0 is a mutated ubiquitin that all lysines are mutated to arginines and only mediates mono-ubiquitination. The nucleoproteins were extracted from S2 cells co-overexpressing several designed molecules and were then used for Co-IP analysis with anti-Myc tag antibody. (**G, H**) ChIP-qPCR assays in *Fzr*-overexpressing S2 cells by using anti-H2Bub antibody (G) and anti-Fzr antibody (H) revealed that the amounts of ubiquitinated H2B and Fzr that binds to the promoter region of the *Myc* gene were elevated after *Fzr* overexpression. Data are presented as mean ± SE (error bars). For the significance test: *P < 0.05, **P < 0.01 versus control. OE, overexpression. AEL, after egg laying.

Given that Fzr/Cdh protein contains seven conserved WD40 domains that are involved in protein–protein interaction but has no domain that is responsible for transcriptional regulation (9,50–52), we further aimed to characterize novel factors that can interact with Fzr to modulate *Myc* transcription. A Co-IP experiment following LC–MS/MS analysis in human HEK293-FT cells identified that Fzr could potentially interact with a total of 641 proteins (Supplementary Table S3). Interestingly, histone H2B is included in this collection of potential Fzr-interacting partners. H2B is a fundamental structural component of chromatin and H2B ubiquitination is essential for gene transcription activation or silencing by affecting chromatin architecture (53–55). We also observed that *Fzr* knockdown in *Drosophila* salivary glands also caused irregular chromatin condensation and this phenotype could not be rescued by *Myc* overexpression (Supplementary Figure S5A–C), further indicating a potential interaction between Fzr and chromatin components. Moreover, a canonical Co-IP experiment by using total proteins from in *Drosophila* S2 cells co-overexpressing both *Drosophila Fzr* and V5-tagged H2B demonstrated that H2B could be co-immunoprecipitated with Fzr (Figure 4A). Additional Co-IP experiment based on the nucleoproteins isolated from S2 cells with *Fzr* overexpression further confirmed that compared to the control, overexpressed Fzr interacted with more chromatin-associated H2B (Figure 4B). Furthermore, *in vitro* GST-pull down assay showed a direct interaction between Fzr and H2B (Figure 4C). Taken together, these observations indicate that Fzr can interact with H2B and affect chromatin structure.

Ubiquitinated H2B (H2Bub) is preferentially located in transcriptionally active chromatin (53,54). We next asked whether the interaction between Fzr and H2B in *Drosophila* mediates H2B ubiquitination. Strikingly, *Fzr* overexpression in *Drosophila* S2 cells promoted H2B ubiquitination (Figure 4D) while *Fzr* knockdown in *Drosophila* salivary glands decreased the H2Bub level (Figure 4E). Furthermore, we performed *in vivo* ubiquitination assays in S2 cells by overexpressing either intact ubiquitin (Ub) or mutated Ub-K0 (only mediates mono-ubiquitination) and found that compared to the control without *Fzr* overexpression, following *Fzr* overexpression, a single stronger signal band of H2Bub was detected in the nucleoproteins with the overexpression of either Ub or Ub-K0 (Figure 4F and Supplementary Figure S7A), revealing that Fzr promotes H2B mono-ubiquitination.

To examine the relationship between H2B ubiquitination and the transcription of *Myc* as a downstream effector of Fzr, we conducted ChIP-qPCR analysis in S2 cells and observed that *Fzr* overexpression enhanced the accumulation of both H2Bub and Fzr within different regions of the potential promoter of only *Myc* gene, but not other selected genes as control, including *CycB*, *HSP90* and *Actin5c* (Figure 4G, H and Supplementary Figure S6A–C). In addition, given that Fzr can activate the E3 ubiquitin ligase activity of APC/C complex to mediate the degradation of their substrates (2,3), we further performed salivary gland-specific knockdown of different subunits of APC/C complex and found that *APC3* knockdown phenocopied the defects caused by *Fzr* knockdown (Supplementary Figure S7B–G), and decreased the H2Bub level (Supplementary Figure S7H), indicating that Fzr promotes H2B ubiquitination through, at least partially, APC3. Taken together, these data demonstrate that Fzr directly interacts with the chromatin-associated H2B to mediate mono-ubiquitination of H2B within the promoter of the *Myc* gene, which is likely involved in orchestrating *Myc* transcription.

### Myc positively regulates the transcription of *MCM6* involving in DNA replication

**Figure 5.**
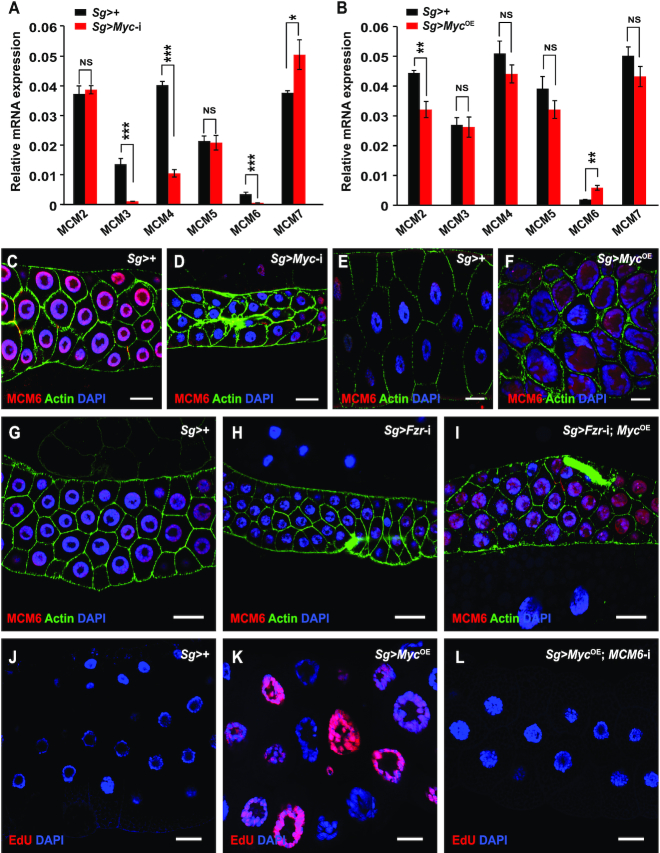
Myc promotes the transcription of the *MCM6* gene involving in DNA replication. (**A**, **B**) Effects of *Myc* expression change on the transcription of all members of the MCM complex that function as components of preRCs and to initiate DNA replication. RT-qPCR analysis showed that the transcription of the *MCM6* gene was decreased after *Myc* knockdown (A) but was increased following *Myc* overexpression (B) in the salivary glands at 96 h AEL. (**C–F**) Immunostaining analysis confirmed that Myc promoted the expression of the MCM6 protein. The MCM6 protein level in the salivary glands was downregulated after *Myc* knockdown at 96 h AEL (C, D) but was upregulated following *Myc* overexpression at 120 h AEL (E, F). (**G–I**) *Fzr* knockdown reduced MCM6 expression in the salivary glands and this reduction was diminished by simultaneous *Myc* overexpression at 96 h AEL. **(J-L)***Myc* overexpression-induced enhancement in DNA replication was impaired by *MCM6* knockdown in salivary glands at 120 h AEL. Data are presented as mean ± SE (error bars). For the significance test: *P < 0.05, **P < 0.01, ***P < 0.001 versus control. OE, overexpression. AEL, after egg laying. Scale bar, 30 μm.

Considering that the expression of the transcription factor Myc in the salivary gland promotes DNA replication in endocycling cells, we further investigated whether Myc regulates the transcription of the six members of the MCM complex that function as components of the preRCs to initiate DNA replication during endocycling progression. RT-qPCR analysis showed that the transcriptions of *MCM3* and *MCM6* were significantly decreased to almost undetectable levels following *Myc* knockdown in the salivary gland (Figure 5A). But, only *MCM6* transcription, not *MCM3* transcription, was significantly promoted by *Myc* overexpression in the salivary glands (Figure 5B), indicating that Myc is involved in the regulation of *MCM6* transcription.

We further assessed the regulatory effect of Myc on MCM6 expression by immunostaining with an antibody against *Drosophila* MCM6. As shown in Figure 5C and D, MCM6 expression was strongly downregulated at the protein level in salivary glands with *Myc* knockdown. Conversely, MCM6 protein expression was increased in the salivary glands after *Myc* overexpression (Figure 5E and F). Epistatic analysis revealed that *Fzr* knockdown-induced reduction of MCM6 expression in the salivary gland was diminished by simultaneous *Myc* overexpression (Figure 5G–I). In addition, salivary gland-specific knockdown of the *MCM6* gene abrogated DNA replication (Supplementary Figure S8A and B), and *Myc* overexpression-induced increase in DNA content as well as enhancement in DNA replication was impaired by *MCM6* knockdown in the salivary glands at 120 h AEL (Figure 5J–L and Supplementary Figure S8C and D). These data suggest that Myc promotes DNA replication in the endocycling salivary gland cells by positively regulating *MCM6* expression.

### Myc directly binds to specific motifs within the promoters of the *CycB* and *MCM6* genes

**Figure 6.**
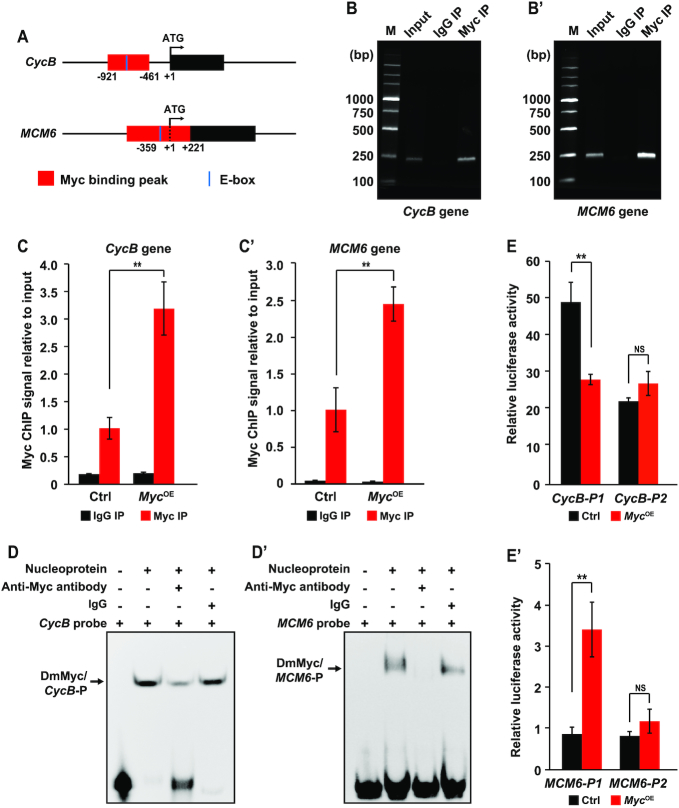
Myc regulates the transcription of the *CycB* and *MCM6* genes by directly binding to specific motif within their promoters. (**A**) Schematic diagram of the Myc binding peaks and potential E-box motifs for Myc binding within the promoters of the *CycB* and *MCM6* genes. One binding peak is located in the region from -921 to -461 within the promoter upstream of the translational start site of the *CycB* gene and one potential E-box for Myc binding is located within this region from -838 to -833. One binding peak exists within the region from –359 to +221 around the translational start site of the *MCM6* gene and there is one potential E-box within this region from –83 to –78. (**B–C′**) ChIP-PCR and ChIP-qPCR assays verified the direct binding of Myc to the promoters of the *CycB* and *MCM6* genes in *Drosophila* salivary glands (B, B’) and S2 cells with *Myc* overexpression (C, C’). (**D, D′**) Electrophoretic mobility shift assay (EMSA) confirmed that Myc can directly bind to specific E-box motifs for Myc binding within the promoters of the *CycB* (D) and *MCM6* genes (D’). Anti-Myc antibody competitively impaired the binding of Myc to the probes targeting E-box motifs. (**E, E′**) Luciferase reporter analyses revealed that Myc inhibited and promoted activities of the *CycB* promoter (E) and the *MCM6* promoter (E’), respectively. P1, complete promoters of the *CycB* and *MCM6* genes containing potential E-box. P2, truncated promoters of the *CycB* and *MCM6* genes without E-box. Data are presented as mean ± SE (error bars). For the significance test: **P < 0.01 versus control. OE, overexpression.

Myc belongs to the bHLH-Zip transcription factor family and regulates the transcription of its downstream targets by specifically binding to conserved cis-regulatory elements such as E-box or E-box-like motifs (49,56,57). We therefore characterized the probability of Myc binding to the promoters of the *Drosophila CycB* and *MCM6* genes. By analysing the *Drosophila* Myc ChIP-Seq data from previous study (49), we found one binding peak within the promoter regions of both *CycB* and *MCM6* (Figure 6A). MatInspector program-based bioinformatics analysis predicted one potential E-box motif for Myc binding within these binding peaks (Figure 6A). Subsequent ChIP-PCR examination in the salivary glands revealed that the DNA regions covering E-box motif within the Myc binding peaks were detectable in the products precipitated with anti-Myc antibody by using specific primers covering E-box motifs (Figure 6B–B′). Similarly, ChIP-qPCR experiment in *Drosophila* S2 cells also confirmed that comparing to the control, the amounts of Myc binding within the peaks were increased following *Myc* overexpression (Figure 6C–C′). Furthermore, we designed biotinylated probes targeting the predicted E-box motifs for Myc binding within the promoters of the *CycB* and *MCM6* gene and performed EMSA experiments. The results showed that the nucleoproteins from S2 cells overexpressing *Myc* could bind to the biotinylated probes in a dose-dependent manner, and this binding was competitively attenuated not only by the unlabeled cold probes (Supplementary Figure S9A and B), but also by specific anti-Myc antibody (Figure 6D–D′). Our results together suggest that Myc directly binds to specific E-box motifs within the promoter regions of both *CycB* and *MCM6*.

We further performed luciferase reporter assay in *Drosophila* S2 cells to examine the effects of Myc on the activity of the promoters of the *Drosophila CycB* and *MCM6* genes. We observed that compared to the control, *Myc* overexpression decreased and increased the activities of *CycB* promoter and *MCM6* promoter, which (P1) contain E-box motifs for Myc binding, respectively (Figure 6E–E′). However, *Myc* overexpression has no effect on the activities of the truncated promoters (P2) that contain no potential E-box motifs (Figure 6E–E′). Taken together, we proposed that Myc regulates the transcription of the *CycB* and *MCM6* genes by directly binding to specific E-box motifs within their promoters.

### The Fzr-H2Bub-Myc signaling cascade is conserved between insect and mammalian cells

**Figure 7.**
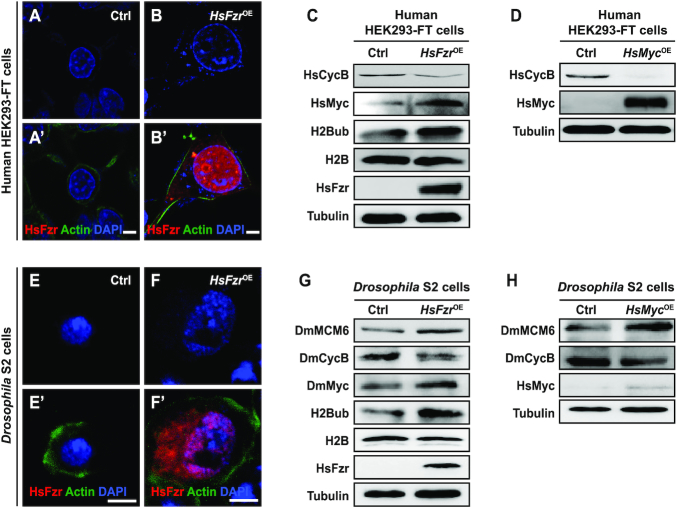
The Fzr-H2Bub-Myc signaling cascade is conserved between insect and mammalian cells. (**A–C**) Effects of the overexpression of the human *Fzr* (*HsFzr*) gene in human HEK293-FT cells. *HsFzr* overexpression not only increased the size of the cell and nucleus (A-B’), but also inhibited HsCycB expression and promoted both HsMyc expression and H2B ubiquitination (C), which is similar to the effects of the overexpression of the *Drosophila* Fzr *(DmFzr*) gene in *Drosophila* S2 cells. (**D**) Overexpression of the human *Myc* (*HsMyc*) gene in HEK293-FT cells inhibited HsCycB expression. (**E–F’**) Ectopic overexpression of the *HsFzr* gene in *Drosophila* S2 cells also increased the size of the cell and nucleus. (**G**) Ectopic overexpression of human *HsFzr* in *Drosophila* S2 cells inhibited DmCycB expression, promoted DmMCM6 expression, and enhanced both DmMyc expression and H2B ubiquitination. (**H**) Ectopic overexpression of human *HsMyc* in S2 cells inhibited DmCycB expression and promoted DmMCM6 expression. OE, overexpression. *Hs*, *Homo sapiens*; *Dm*, *Drosophila melanogaster*. Scale bar, 5 μm.

We sought to evaluate whether the Fzr-H2Bub-Myc signaling cascade involved in endoreplication is conserved in insects and mammals. Intriguingly, overexpression of the human homolog (*HsFzr*) of *Drosophila Fzr* (*DmFzr*) in human embryonic kidney-derived HEK293-FT cells phenocopied the effects of *DmFzr* overexpression on increasing DNA content and cell size (Figure 7A–B′). Notably, we also found that in HEK293-FT cells, overexpression of either *HsFzr* or *HsMyc* dramatically reduced HsCycB expression (Figure 7C and D). In addition, *HsFzr* overexpression increased HsMyc expression and the level of H2Bub (Figure 7C). These data suggest that the Fzr-H2Bub-Myc signaling cascade also participates in DNA replication in mammalian cells.

We further performed a cross-species analysis to examine the functional conservation of the Fzr-H2Bub-Myc signaling cascade. Our data revealed that, similar to *DmFzr* overexpression in *Drosophila* S2 cells (Supplementary Figure 1F), overexpression of either *HsFzr* or *BmFzr* from the lepidopteran insect *Bombyx mori* in *Drosophila* S2 cells also enlarged the nuclei, implying cross-species conservation of Fzr function in endoreplication (Figure 7E-F’ and Supplementary Figure S10A). Consistent with this result, DmCycB expression was significantly reduced, while both DmMCM6 expression and the level of H2Bub were enhanced following overexpression of either *HsFzr* or *BmFzr* (Figure 7G and Supplementary Figure S10B). Furthermore, *HsMyc* and *BmMyc* overexpression in S2 cells decreased DmCycB expression and increased DmMCM6 expression (Figure 7H and Supplementary Figure S10C). Finally, Co-IP assays showed that both HsFzr and BmFzr interact with *Drosophila* H2B (Supplementary Figure S10D-E). These results suggest that the involvement of the Fzr-H2Bub-Myc signaling cascade in endoreplication progression is conserved between insect and mammalian cells.

## DISCUSSION

Fzr/Cdh1, a WD40 domain-containing protein, has been demonstrated to control the transition from the mitotic cycle to the endocycle and is required for endoreplication in *Drosophila* and mammals (2,3). Here, we outlined a new mechanism of Fzr-mediated regulation of endoreplication progression in *Drosophila* salivary gland in which Fzr interacts with histone H2B to mediate H2B ubiquitination and stimulates *Myc* transcription. The Fzr-H2Bub-Myc signaling cascade is conserved between insect and mammalian cells and is involved in regulating the transcription of cell cycle regulators, *CycB* and *MCM6*, in different manners.

### Fzr is linked with Myc function during endoreplication

Previous studies in *Drosophila* showed that tissue-specific inhibition of *Fzr* expression via RNAi in either follicle cells of the adult ovary or cells of the larval prothoracic gland blocks endocycle progression (9,13,58) and that *Fzr* mutation inhibits endoreplication in the salivary gland (8). Moreover, Myc, which is defined as both a transcription factor and an oncogene, has long been implicated in regulating various cellular processes, including growth, proliferation, differentiation, and oncogenesis (57,59). *Myc* mutation in *Drosophila* leads to the arrest of growth and endoreplication in ovarian follicle cells and salivary gland cells, which undergo endoreplication (60,61). However, whether Fzr can link with Myc to mediate endoreplication in endoreplicating cells remains unclear. Our data revealed that salivary gland-specific knockdown of either *Fzr* or *Myc* results in similar defects, including inhibition of endoreplication; decrease of gland size; activation of *CycB* transcription, which is absent in endocycling cells; and downregulation of *MCM6* transcription, which is involved in DNA replication. We also found that *Fzr* knockdown in the salivary glands decreases the transcription of the *Myc* gene, and the defects resulting from *Fzr* knockdown can be partially ameliorated by *Myc* overexpression. Therefore, we conclude that Fzr is linked with Myc to regulate the progression of endoreplication in the *Drosophila* salivary gland.

### Fzr promotes H2B ubiquitination to regulate *Myc* transcription

A striking finding of our study is that Fzr interacts with chromatin-associated histone H2B to promote H2B ubiquitination, which in turn directly regulates *Myc* transcription. Previous studies reported that Fzr activates the APC/C complex, which has E3 ubiquitin ligase activity, and that high APC/C^Fzr^ activity induces the ubiquitination and subsequent degradation of direct substrates, such as Geminin, ORC proteins, CycB, and Nek2 kinase, in endocycling *Drosophila* cells (14,15,24,25). Upregulation of *Fzr* expression in follicle cells of the adult ovary, which are undergoing mitotic cycling, causes precocious endocycling and reduces the levels of CycB and CycA (13). Additional evidence revealed that Fzr directly interacts with APC/C^Fzr^ substrates and subsequently ubiquitylates them (36,62). Intriguingly, by combining a Co-IP experiment and an *in vivo* ubiquitination assay in *Drosophila* salivary gland and S2 cells, we found that Fzr can directly interact with chromatin-associated H2B and promote H2B ubiquitination, and the downregulation of the APC3 subunit of the APC/C complex not only phenocopies the effects of *Fzr* knockdown on salivary gland endoreplication but also decreases the H2Bub level. Previous studies also reported that H2B can be ubiquitinated by the E3 ubiquitin ligase Bre1 in yeast or its homologous RNF20/RNF40 complex in mammals (53,55,63), and *Bre1* mutation in *Drosophila* reduces H2B ubiquitination-dependent H3K4 methylation (64). Therefore, further investigations are needed to address the detailed mechanisms underlying Fzr regulation of H2B ubiquitination during endoreplication.

H2B ubiquitination is generally involved in activating gene transcription by regulating chromatin organization and subsequently mediating transcription initiation and elongation (55,65,66). Increasing evidence indicates that H2Bub is preferentially enriched in the promoters of actively transcribed genes (66–69). Notably, we found that *Fzr* overexpression enhanced H2Bub accumulation in the region upstream of the translation start site of the *Myc* gene, a downstream effector of Fzr. Taken together, our findings suggest that Fzr-mediated H2B ubiquitination likely stimulates *Myc* transcription and that the Fzr-H2Bub-Myc signaling cascade is involved in endoreplication in *Drosophila* salivary gland.

### The Fzr-H2Bub-Myc signaling cascade regulates the transcription of cell cycle regulators

Endoreplication mainly involves three biological events, namely, the mitotic-to-endocycle transition, oscillation of DNA re-replication, and progression of DNA replication (2). The Fzr-H2Bub-Myc signaling cascade has been found here to be involved in regulating the transcription of two cell cycle regulators involved in endoreplication in the *Drosophila* salivary gland—CycB, absent during mitotic-to-endocycle transition, and MCM6, associated with DNA replication. CycB is required for the G2/M transition during mitosis (21–23,70), and activation of CycB/CDK1 may paticipate in the inhibition of endoreplicaiton progression (8). Together with findings from a previous report indicating that the CycB protein is not expressed in the *Drosophila* salivary gland and that *CycB* mRNA is not expressed in the *Bombyx* silk gland (8,26), our observation that the expression of both *CycB* mRNA and CycB protein was induced following the downregulation of either *Fzr* or *Myc* suggests that the Fzr-H2Bub-Myc signaling cascade transcriptionally inhibits *CycB* expression during endoreplication.

The MCM complex, comprising the structurally related MCM2–7 subunits, is loaded on replication origins during G1 phase to initiate DNA synthesis and elongate the DNA strands in eukaryotic cells (30,71). Downregulation of *MCM4* and *MCM7* expression blocks endocycle progression in endocycling fat body cells in migratory locusts by inhibiting DNA replication (33). The interaction between MCM6 and the DNA replication factor Cdt1 during G1 phase serves as a platform for the formation of preRCs in the nucleoplasm and the loading of the complex on replication origins (72,73); abolishing this interaction prevents the formation of MCM heterohexamers and subsequently inhibits DNA synthesis (73–75). We found that the Fzr-H2Bub-Myc signaling cascade positively regulates transcription of the *MCM6* gene in the *Drosophila* salivary gland. Given that Fzr controls endoreplication entry and MCM function in DNA replication, our results indicate a link between endoreplication entry and DNA replication in endocycling cells.

The transcription factor Myc regulates the transcription of downstream targets by specifically binding to conserved E-box sequences in both mammals and *Drosophila* (49,56). Our results show that Myc directly binds to specific motifs within the promoters of the *Drosophila CycB* and *MCM6* genes, which then negatively and positively regulates the transcription of *CycB* and *MCM6*, respectively. In fact, some transcription factors can positively and negatively regulate the transcription of downstream targets by binding to cis-regulatory elements within the promoters of the targeting genes, like Kr-h1 in insect (76) and Dmrt1 in fish (77,78). Mechanistically, transcription factors generally need to recruit and interact with some co-activators and/or co-repressors to mediate transcriptional regulation (79). Thus, it will be of interest to identify co-activators and co-repressors involving in Myc regulation on the transcription of the *CycB* and *MCM6* genes during endoreplication.

## DATA AVAILABILITY

All raw data of transcriptome change following *Fzr* overexpression have been deposited with the Sequence Read Archive of the National Center for Biotechnology Information (NCBI) database under accession number PRJNA509304.

## Supplementary Material

gkaa158_Supplemental_Files
