# Supplementary material for: A novel transcriptional cascade is involved in Fzr-mediated endoreplication
Source: Nucleic Acids Res. 2020 Mar 17;48(8):4214–29. doi: 10.1093/nar/gkaa158 (PMC7192621; doi:10.1093/nar/gkaa158)
Supplement: gkaa158_Supplemental_Files [file gkaa158_supplemental_files.zip › Supplementary Figures.pdf]

Supplementary Figures

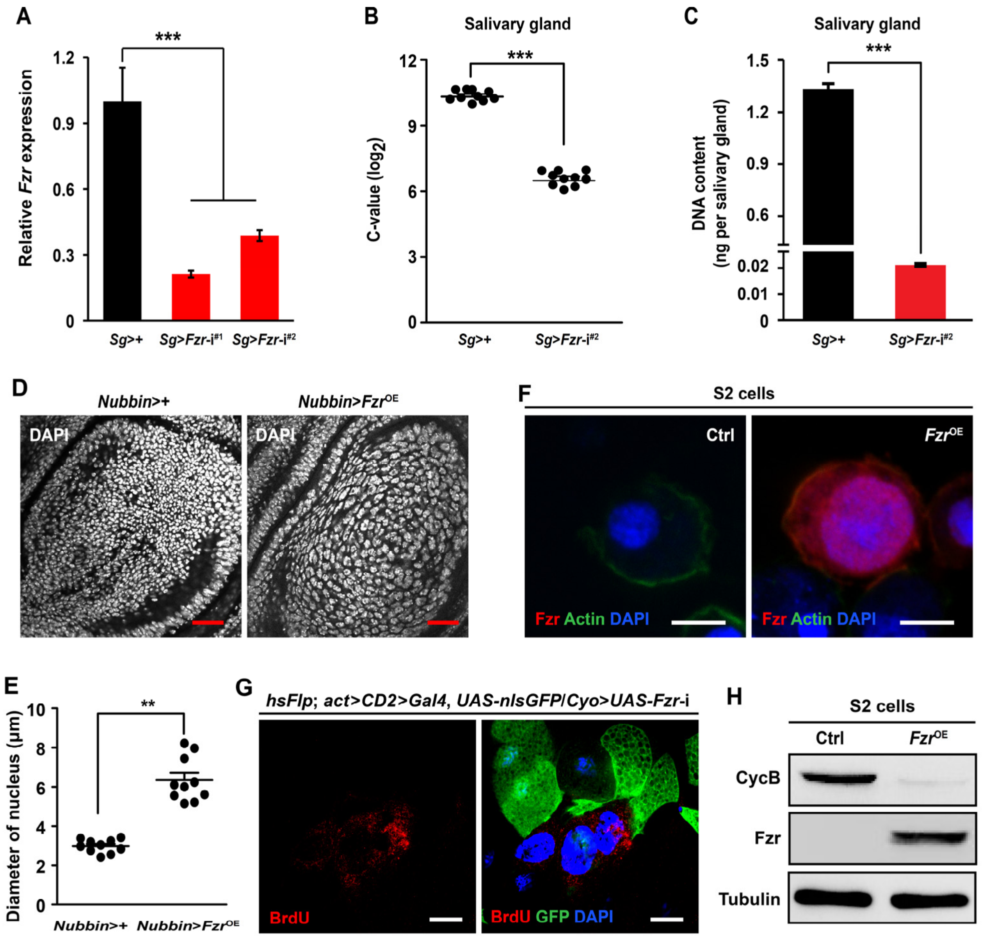

Supplementary Figure S1. Changes of *Fzr* expression affect endoreplication progression and CycB expression.

(A) The efficiency of salivary gland-specific knockdown mediated by two RNAi lines targeting different sequence regions of the *Fzr* gene. *Fzr*-i$^{\#1}$, *UAS-Fzr* RNAi from VDRC (#V25550); *Fzr*-i$^{\#2}$, *UAS-Fzr* RNAi from TsingHua Fly Center (#TH2015000745.S). **(B-C)** *Fzr*-i$^{\#2}$-based knockdown of the *Fzr* gene in the salivary glands resulted in a significant reduction in the C-value (B) and DNA content (C). **(D-E)** *Fzr* overexpression driven by *Nubbin*-Gal4 in *Drosophila* wing disc increased the size of the nuclei. Scale bar, 30 μm. **(F)** *Fzr* overexpression in *Drosophila* S2 cells increased the size of the cells and nuclei. Scale bar, 5 μm. **(G)** Mosaic analysis revealed that RNAi-mediated *Fzr* knockdown in *Drosophila* salivary glands inhibited DNA replication. Scale bar, 30 μm. **(H)** *Fzr* overexpression in S2 cells decreased the level of the CycB protein. Data are presented as mean ± SE (error bars). For the significance test: $^{**}P < 0.01$, $^{***}P < 0.001$ vs. control. OE, overexpression.

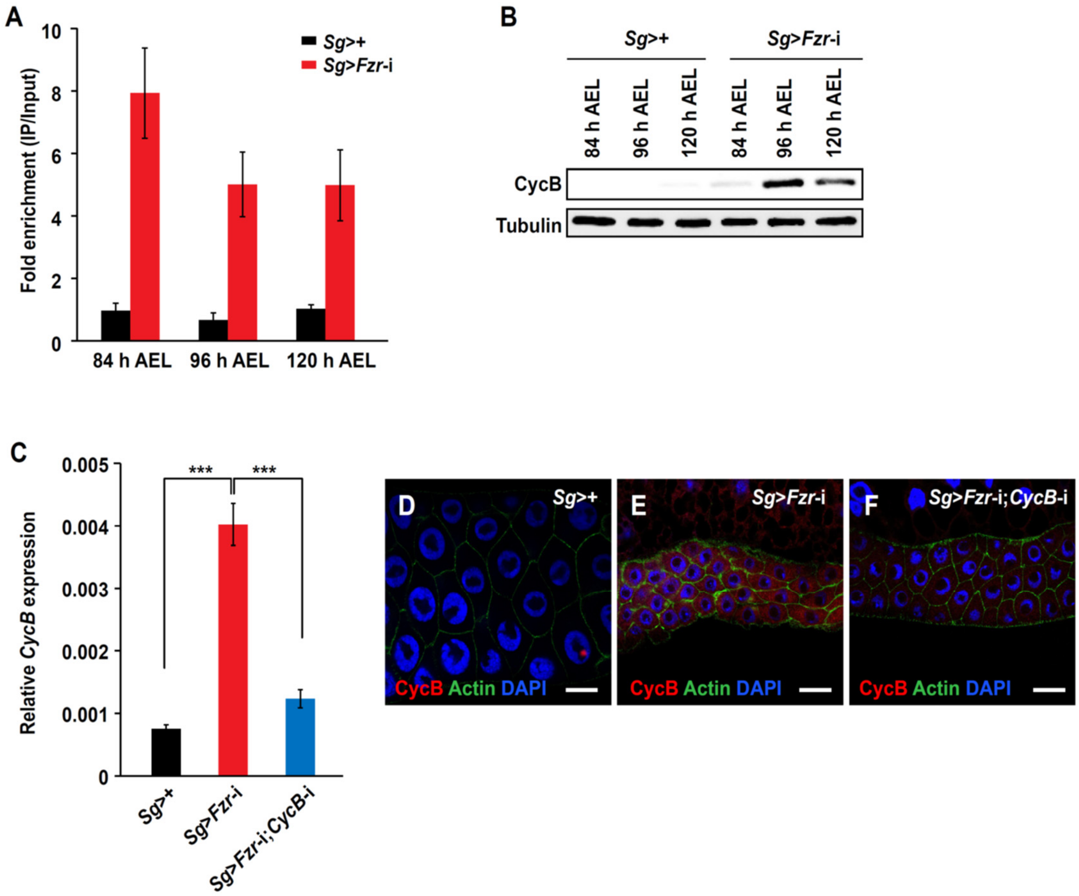

Supplementary Figure S2. Increased *CycB* mRNA following *Fzr* knockdown in the salivary glands is a driving force behind the increased CycB protein levels.

(A) Translating ribosome affinity purification (TRAP) analysis following RT-qPCR showed that the increased *CycB* mRNA was highly translated at 84 h AEL in the salivary glands with *Fzr* knockdown and exhibited a moderate translation at 96 h AEL and 120 h AEL. **(B)** CycB proteins were accumulated from 84 h AEL to 96 h AEL in the salivary glands with *Fzr* knockdown and were detectable at 120 h AEL. **(C)** The accumulation of the *CycB* mRNA in the salivary glands with *Fzr* knockdown was abrogated by *CycB* knockdown at 96 h AEL. **(D-F)** *CycB* knockdown in the salivary glands also impaired *Fzr* knockdown-induced accumulation of the *CycB* proteins at 96 h AEL. Data are presented as mean ± SE (error bars). For the significance test: ****P* < 0.001 vs. control. OE, overexpression. AEL, after egg laying. Scale bar, 30 μm.

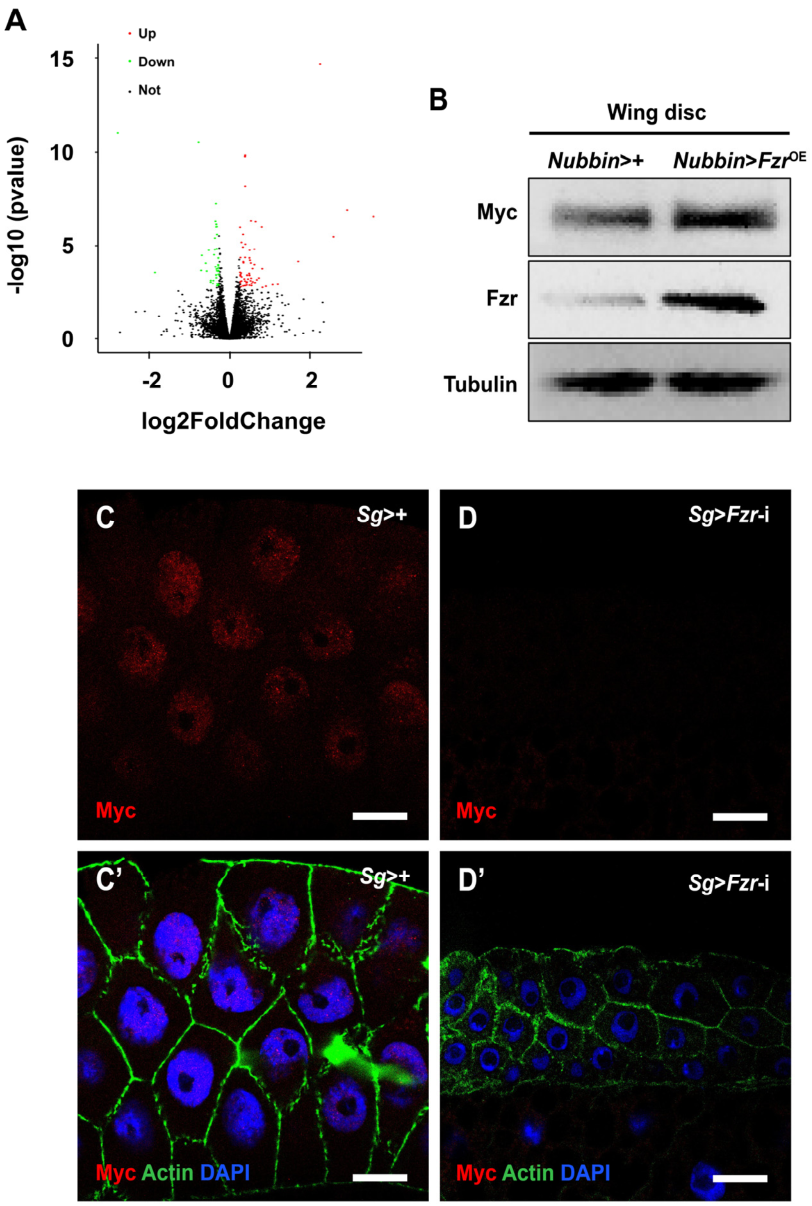

Supplementary Figure S3. Fzr promotes the expression of the *Myc* gene.

(A) Transcriptome sequencing revealed that *Fzr* overexpression in *Drosophila* S2 cells significantly upregulated and downregulated the transcription of 38 and 39 genes, respectively. The *Myc* gene was included in the list of the up-regulated genes. **(B)** Western blotting revealed that *Fzr* overexpression driven by *Nubbin*-Gal4 in wing disc promoted the expression of Myc. **(C-D')** *Fzr* knockdown in the salivary glands reduced Myc protein expression at 96 h AEL. OE, overexpression. AEL, after egg laying. Scale bar, 30 μm.

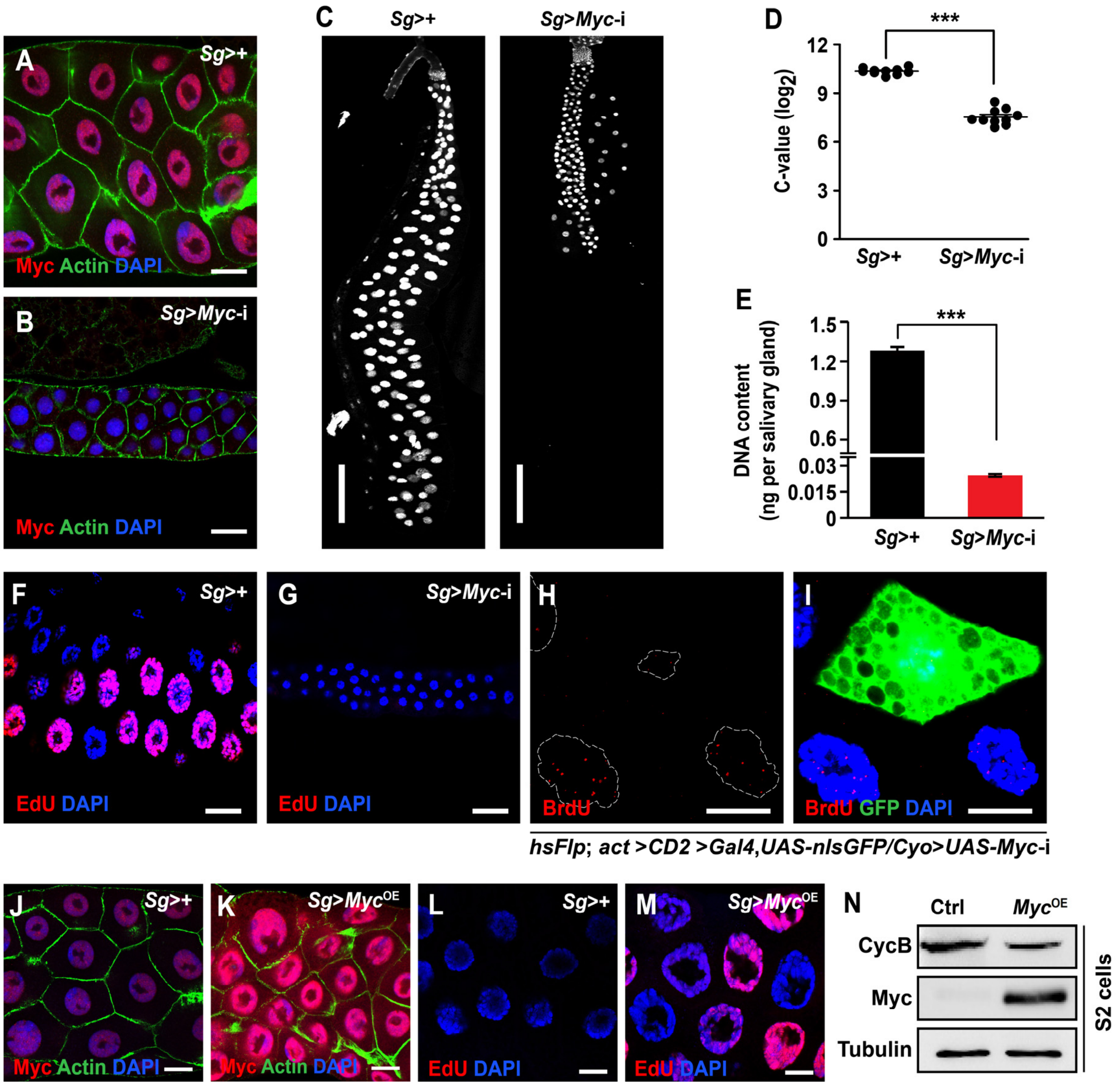

Supplementary Figure S4. Changes of *Myc* expression affect endoreplication progression and CycB expression.

(A-E) Effects of salivary gland-specific *Myc* knockdown. The efficiency of *Myc* knockdown in the salivary glands was detected at 96 h AEL (A-B). Scale bar, 30 μm. Immunostaining analysis showed that following *Myc* knockdown in the salivary glands, gland size (C), C-value (D), and DNA content (E) were reduced at 120 h AEL, compared to the control. Scale bar, 180 μm. **(F-G)** EdU staining showed that *Myc* knockdown in the salivary glands inhibited DNA replication at 96 h AEL. Scale bar, 30 μm. **(H-I)** Mosaic assay revealed that compared to the cells as control in the salivary glands, clonal knockdown of the *Myc* gene reduced DNA replication, revealing by BrdU staining. Scale bar, 30 μm. **(J-M)** Effects of salivary gland-specific *Myc* overexpression on DNA replication**.** The efficiency of *Myc* overexpression in the salivary glands was detected at 120 h AEL (J-K). EdU staining showed that

DNA replication was enhanced following *Myc* overexpression in the salivary glands at 120 h AEL (L-M). Namely, when DNA replication had been stopped in the salivary glands of the control at 120 h AEL (L), the salivary glands with *Myc* overexpression were still undergoing strong DNA replication (M), which can be labelled with EdU. Scale bar, 30 μm. (**N**) *Myc* overexpression in *Drosophila* S2 cells decreased CycB expression. Data are presented as mean ± SE (error bars). For the significance test: ****P* < 0.001 vs. control. OE, overexpression. AEL, after egg laying.

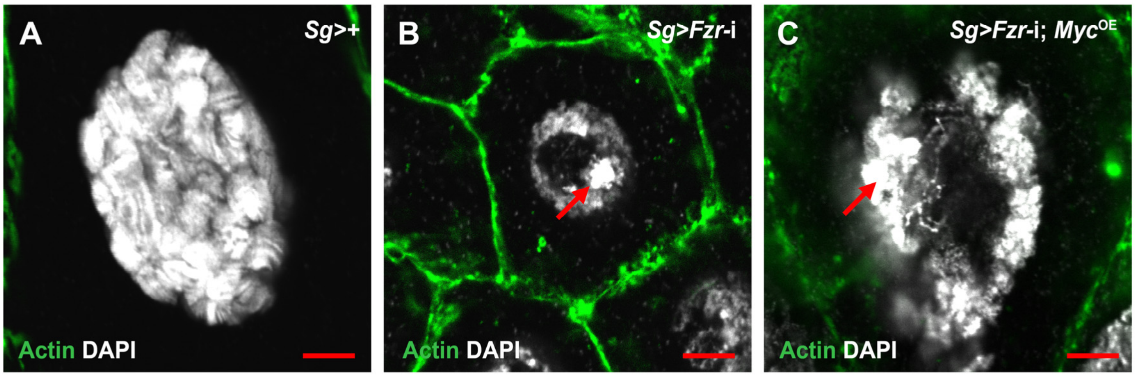

Supplementary Figure S5. Fzr affects the morphological structure of polytene chromosomes. **(A-B)** *Fzr* knockdown in the salivary glands altered the morphological structure of polytene chromosomes with an undistinguishable banding pattern and obvious chromosome condensation. **(C)** *Fzr* knockdown-induced abnormal polytene chromosomal structure was not rescued by *Myc* overexpression. Scale bar, 5 μm.

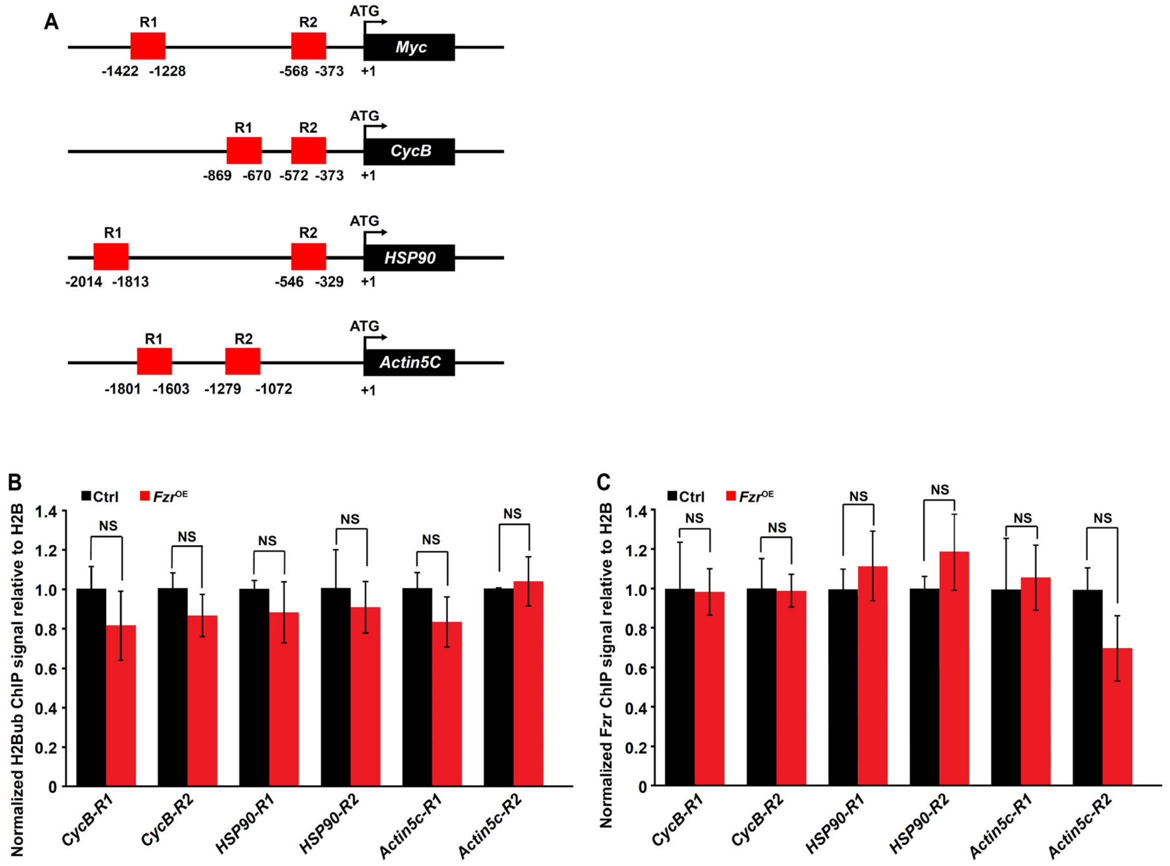

Supplementary Figure S6. ChIP-qPCR assays in *Fzr*-overexpressing *Drosophila* S2 cells.

(A) Schematic diagram of the specific regions for ChIP-qPCR examination. For each gene, two primer pairs covering the different regions (R1 and R2) within the promoter upstream of the translational start site were designed and used for qPCR detection. **(B-C)** *Fzr* overexpression could not change the accumulation of H2Bub (B) and Fzr (C) within the different regions of the promoters of both *CycB* and other housekeeping genes (*Hsp90* and *Actin5C*). Data are presented as mean ± SE (error bars). OE, overexpression.

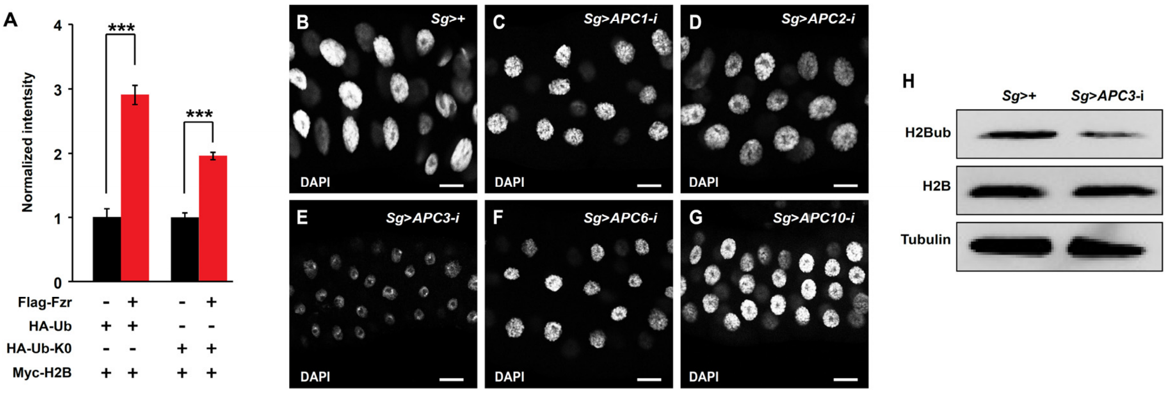

Supplementary Figure S7. Fzr promotes H2B mono-ubiquitination through APC3.

(A) The normalized intensity of HA-tagged H2Bub relative to Myc-tagged H2B in the products of Co-IP from *Drosophila* S2 cells with anti-Myc tag antibody, based on Figure 4F. The results revealed that *Fzr* overexpression in S2 cells promoted H2B mono-ubiquitination. **(B-G)** Salivary gland-specific knockdown of different subunits of the APC/C complex. Only *APC3* RNAi can phenocopy the effect of *Fzr* knockdown on the size of gland and nuclei. **(H)** *APC3* knockdown in *Drosophila* salivary gland decreased the H2Bub level. Data are presented as mean ± SE (error bars). For the significance test: ***P < 0.001 vs. control. Scale bar, 30 μm.

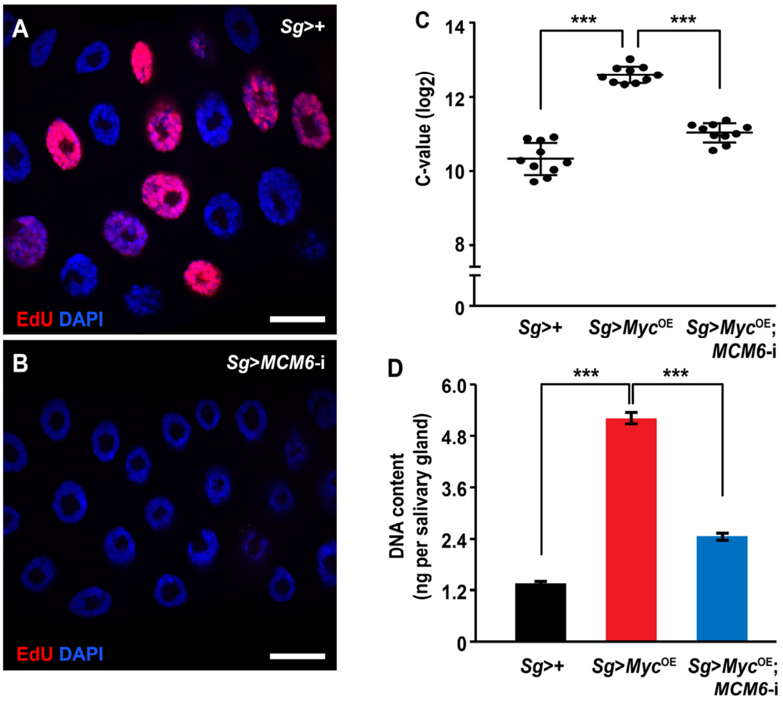

Supplementary Figure S8. Myc regulates the endoreplication of salivary gland through MCM6. **(A-B)** Salivary gland-specific knockdown of the *MCM6* gene abrogated DNA synthesis at 96 h AEL. **(C-D)** Epistasis analysis in the salivary gland revealed that *MCM6* knockdown in the salivary gland reduced the effects of *Myc* overexpression on C-value (C) and DNA content (D) at 120 h AEL. Data are presented as mean ± SE (error bars). For the significance test: ****P* < 0.001 vs. control. OE, overexpression. AEL, after egg laying. Scale bar, 30 μm.

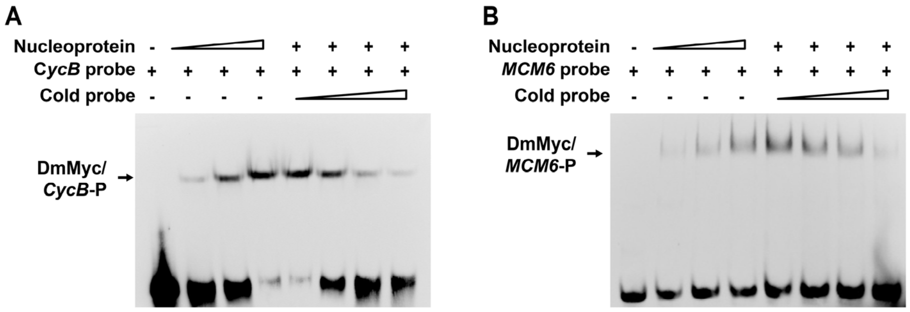

Supplementary Figure S9. Myc directly binds to specific motifs within the promoters of the *CycB* and *MC*M6 genes in a dose-dependent manner.

EMSA confirmed that Myc could bind to the biotinylated probes covering specific E-box motifs within the promoters of the *CycB* (A) and *MCM6* (B) genes in a dose-dependent manner, and this binding was competitively repressed by the unlabelled cold probes.

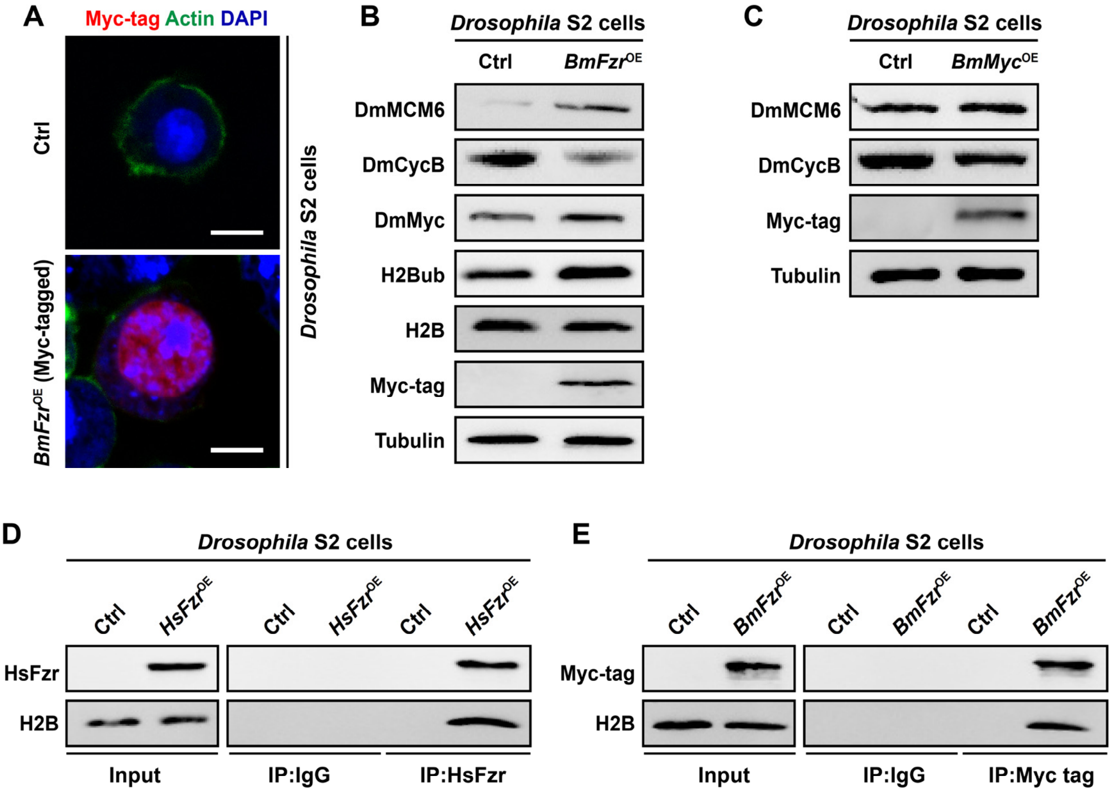

Supplementary Figure S10. *Bombyx* and human Fzr proteins can interact with *Drosophila* H2B. **(A)** Ectopic overexpression of the Myc-tagged *Bombyx mori Fzr* (*BmFzr*) gene in *Drosophila* S2 cells increased the size of the cells and nuclei, indicating that the cells enter endoreplication after *Fzr* overexpression. Scale bar, 5 μm. **(B)** In S2 cells, ectopic *BmFzr* overexpression inhibited *Drosophila* DmCycB expression, promoted DmMCM6 expression, and enhanced both DmMyc expression and H2B ubiquitination. **(C)** In S2 cells, ectopic overexpression of *BmMyc* inhibited DmCycB expression and promoted DmMCM6 expression. **(D-E)** Both human HsFzr and BmFzr can interact with *Drosophila* DmH2B. *HsFzr* and *BmFzr* were separately overexpressed in S2 cells. At 48 h after transfection, cells were lysed and a Co-IP assay was performed. *Bm*, *Bombyx mori*; *Dm*, *Drosophila melanogaster*; *Hs*, *Homo sapiens*. OE, overexpression.
