# Supplementary material for: A novel transcriptional cascade is involved in Fzr-mediated endoreplication
Source: Nucleic Acids Res. 2020 Mar 17;48(8):4214–29. doi: 10.1093/nar/gkaa158 (PMC7192621; doi:10.1093/nar/gkaa158)
Supplement: gkaa158_Supplemental_Files [file gkaa158_supplemental_files.zip › Supplementary Table S1.pdf]

Supplementary Table S1. The primers used in this study

Purpose	Primer name	Primer sequence
RT-qPCR	*CycB*	Forward: 5' GTTCCGACCAAGACCACTGT 3'
		Reverse: 5' TCTTTGCGGGAAACAGCTAC 3'
	MCM2	Forward: 5' GGACAAGATCGCCAAGATGT 3'
		Reverse: 5' AACTTCTGCGCCTCGATAAA 3'
	MCM3	Forward: 5' CTTCGTTATGCTGGACGTGA 3'
		Reverse: 5' CAGGGCATCGTACTTTTCGT 3'
	MCM4	Forward: 5' CATTCTCACCACCGGCTTAT 3'
		Reverse: 5' CCTCATCTTGCACCTCCTTC 3'
	MCM5	Forward: 5' CCGGACATTTAACGAGGAGA 3'
		Reverse: 5' GTGATCTCGTCGGCTACCTC 3'
	MCM6	Forward: 5' GCAGCAGTTCAAGTTCACCAA 3'
		Reverse: 5' ACTCACATCGGGCACCACA 3'
	MCM7	Forward: 5' TGCTCTCACGTTTCGATCTG 3'
		Reverse: 5' GCTCATCCGGAATAGTTGGA 3'
	Myc	Forward: 5' GTCGAGCACACCGTATCAGA 3'
		Reverse: 5' AGATAAACGCTGCTGGAGGA 3'
	Fzr	Forward: 5' TACTCGTTGTCACCCGTCAG 3'
		Reverse: 5' GCGCTCCACAGATAGACACA 3'
Overexpression	*Fzr*	Forward: 5' CCGGAATTCATGTTTAGTCCCGAGTACGAGAAG 3'
		Reverse: 5' AAGGAAAAAAGCGGCCGCTTATCTGATATTGGCAAACAGATTC 3'
	Myc	Forward: 5' CCGGAATTCATGGCCCTTTACCGCTCTG 3'
		Reverse: 5' AAGGAAAAAAGCGGCCGCCTATCCACTAACCGAGCGC 3'
	H2B	Forward: 5' CCGGAATTCATGGAACAAAAACTCATCTCAGAAGAGGATCTGCCTCCGAAAACTAGTGGAA 3'

		Reverse: 5' CCGCTCGAGTTTAGAGCTGGTGTACTTGGTG 3'
	Ubiquitin	Forward: 5' CCGGAATTCATGTACCCCTATGATGTGCCTGACTACGCACAGATCTTCGTGAAGACC 3'
		Reverse: 5' AAGGAAAAAAGCGGCCGCTCAACCACCTCTGAGACGGAG 3'
	BmFzr	Forward: 5' CCGGAATTCATGTTCAGTAAACATTATGA 3'
		Reverse: 5' CCGCTCGAGTTACAGATCTTCTTCAGAAATAAGTTTTTGTTCTCTGAGTGCGGTGTATA 3'
	BmMyc	Forward: 5' CCGGAATTCATGTCGCCGCCGCTAGA 3'
		Reverse: 5' CCGCTCGAGTCACAGATCTTCTTCAGAAATAAGTTTTTGTTCGTACGGGTGGTAGCGA 3'
	HsFzr	Forward: 5' CCGGAATTCATGGACCAGGACTATGAGCG 3'
		Reverse: 5' AAGGAAAAAAGCGGCCGCTTACCGGATCCTGGTGAAGA 3'
	HsMyc	Forward: 5' CCGGAATTCATGGATTTTTTTCGGGTAGTG 3'
		Reverse: 5' CCGCTCGAGTTACGCACAAGAGTTCCGTAG 3'
Prokaryotic expression	*Fzr*	Forward: 5' CCGGAATTCATGTTTAGTCCCGAGTACGAGAAG 3'
		Reverse: 5' CCGCTCGAGTTATCTGATATTGGCAAACAGATTC 3'
	H2B	Forward: 5' CCGCTCGAGATGCCTCCGAAAACTAGTGGAAG 3'
		Reverse: 5' CCGGAATTCTTATTTAGAGCTGGTGTACTTGGTG 3'
EMSA	Biotin-*CycB*	Forward: 5' biotin-GCTGTGCACCAGTACTGGTCACACTAAAACTATCGAAAATTATCGCACGTATCGCATCT 3'
	CycB	Forward: 5' GCTGTGCACCAGTACTGGTCACACTAAAACTATCGAAAATTATCGCACGTATCGCATCT 3'
		Reverse: 5' AGATGCGATACGTGCGATAATTTTCGATAGTTTTAGTGTGACCAGTACTGGTGCACAGC 3'
	Biotin-*MCM6*	Forward: 5' biotin-CAGAAAAAGGCGCCAAAACCGAGCAAAGCACGCAGCTGTAAACATTCGCAA 3'
	MCM6	Forward: 5' CAGAAAAAGGCGCCAAAACCGAGCAAAGCACGCAGCTGTAAACATTCGCAA 3'
		Reverse: 5' TTGCGAATGTTTACAGCTGCGTGCTTTGCTCGGTTTTGGCGCCTTTTTCTG 3'
Promoter cloning	*CycB* P1-F	Forward: 5' CGGGGTACCTATGGGTAGCACTGTTTCAGCC 3'
	CycB P2-F	Forward: 5' CGGGGTACCACTGCGGCTTAAAAGGGAAC 3'
	CycB P1/2-R	Reverse: 5' CCCAAGCTTGCACCGCAAGACTGATCCT 3'
	MCM6 P1-F	Forward: 5' CGGGGTACCTTGCCACCCCCAAGCAACTGTCAG 3'
	MCM6 P2-F	Forward: 5' CGGGGTACCAAACGAAAGCAATCTCGGAAT 3'

	MCM6 P1/2-R	Reverse: 5' TCCCCCGGGTTGGGTCACTTCTTGCTTCAA 3'
H2Bub and Fzr ChIP-qPCR	*Myc*-R1	Forward: 5' AAAAAGTGCCCAACTTGCTG 3'
		Reverse: 5' GTCGTTGCGTTCCGATAAAT 3'
	Myc-R2	Forward: 5' CGGCAGCGATAGCATAAAAT 3'
		Reverse: 5' GATCCTTGCCGGTATTCTGA 3'
	CycB-R1	Forward: 5' AGCACTGTTTCAGCCCTGTC 3'
		Reverse: 5' TCGATGATAAATGCCGATGA 3'
	CycB-R2	Forward: 5' CCCGCACTCGACTTGTAAAT 3'
		Reverse: 5' AGTGTTGTGCCCACCATTTT 3'
	HSP90-R1	Forward: 5' TCAGCTGGTGATATCGATGG 3'
		Reverse: 5' TGGGGAACAAAAATTGGAAA 3'
	HSP90-R2	Forward: 5' ATGCACGCCCATGACTAATA 3'
		Reverse: 5' TCGTGTCTGTGTGAGTGAGCAT 3'
	Actin5C-R1	Forward: 5' GGATACTCCTCCCGACACAA 3'
		Reverse: 5' GCGGCTTTTCTCGATTATTG 3'
	Actin5C-R2	Forward: 5' TTAAAGCCAAGCTCGCTGAT 3'
		Reverse: 5' CGGCTGCCTACGTCACTATT 3'
Myc ChIP-qPCR	*CycB* promoter	Forward: 5' GTAACGTTTTCTTTTTCGGCTAA 3'
		Reverse: 5' AATGCCGATGAGTTTGACGAT 3'
	MCM6 promoter	Forward: 5' CCCAAGCAACTGTCAGAAAA 3'
		Reverse: 5' GGGCGCGTGTACTTGATCTC 3'
In situ hybridization	DIG-*CycB*-T7	Forward: 5' TAATACGACTCACTATAGGGGGCATAAGTCGTCCCATCGC 3'
		Reverse: 5' TAATACGACTCACTATAGGGATCAGCACGGCTCGCATCTT 3'
	DIG-*MCM6*-T7	Forward: 5' TAATACGACTCACTATAGGGAGATTCGCATCCAGGAGACG 3'
		Reverse: 5' TAATACGACTCACTATAGGGTGCCTTGCCCGAGGTGTAGA 3'
